# Situational pathogen avoidance mediates the impact of social connectedness on preventive measures during the COVID-19 pandemic

**DOI:** 10.1038/s41598-023-29239-y

**Published:** 2023-02-10

**Authors:** Frederike Taubert, Philipp Sprengholz, Lars Korn, Sarah Eitze, Marc Wiedermann, Cornelia Betsch

**Affiliations:** 1 Institute for Planetary Health Behavior, Health Communication, University of Erfurt, Erfurt, Germany; 2 Health Communication Working Group, Implementation Research, Bernhard Nocht Institute for Tropical Medicine, Hamburg, Germany; 3 Robert Koch Institute, Berlin, Germany

**Keywords:** Human behaviour, Risk factors

## Abstract

During the COVID-19 pandemic, physical distancing was one of the more important behaviours for reducing the spread of the virus. The present study investigated the influence on pathogen avoidance of familiarity with other people at private gatherings. Based on the social identity model of risk taking and the theory of the behavioural immune system, we assumed that greater familiarity with others would make people feel more connected with one another and decrease situational pathogen avoidance. This could result in lower perceptions of the risk of contracting COVID-19 and fewer protective behaviours. Two experiments (n_1_ = 1022, n_2_ = 994) showed that the negative influence of greater familiarity on the perceived risk of infection and protective behaviour is explained by an increased feeling of connectedness and less feeling of situational pathogen avoidance. In an additional survey, the participants (n = 23,023) rated the quality of their past social contacts. The correlational analyses showed that the familiarity of the other person was more important in explaining variance in protective behaviours than attitudes toward those behaviours or the pandemic situation itself. Understanding the process that result in an explosive increase in infection after social gatherings can improve infection control in the future.

## Introduction

The years 2020 through 2022 were dominated by the COVID-19 pandemic, and waning immunity from infections and immunizations suggested that the subsequent indoor seasons would also bring increased infection numbers. As humans are the hosts of the coronavirus, every social interaction can lead to transmission, but protective behaviours, such as physical distancing, wearing a face mask, and adequate ventilation, can reduce the risk of transmission^1,2^. When vaccination against SARS-CoV-2 was widely unavailable or when uptake was still too low, many governments tried to control the spread of the virus by reducing mass gatherings, e.g., by closing schools or nonessential businesses. Citizens were also asked to reduce private contacts outside their own households, sometimes under force of law. New variants of concern may make this type of behavioural change necessary again in the future.

Complying with the recommendations can protect oneself and others from transmission. It seems plausible that people may be especially motivated to show protective behaviours when interacting with their friends and family, as they want to protect their loved ones, but some psychological theories contradict this assumption. The present contribution shows how familiarity and connectedness can lead to counterintuitively risky behaviour, putting both one’s own and the other person’s health at risk.

The general idea is rooted in social identity theory: people define themselves by being members of various social groups^3–5^. A shared social identity affects thoughts, attitudes, perceptions, and behaviours^6^. Thus, we feel, think, and act differently when we are around people with whom we feel connected. This also affects whether we perceive others as a potential source of risk. Several studies that tested the social identity model of risk taking have shown that sharing a social identity predicts lower perceptions of risk across diverse contexts^7^. For example, participants estimated the risk of infection as being lower when the potential disease vector was an ingroup member as compared to an outgroup member. Moreover, in a field study, participants engaged in more risk behaviour, e.g., staying longer in freezing water at a big event, when the perceived shared identity was greater^7^. In another experiment, beer in a can with colours associated with the ingroup was perceived as safer and less dangerous than beer in a standard can, which led to higher alcohol consumption^8^. Finally, people perceived fewer health risks in mass gatherings where they shared a social identity with the others at the gathering^9^.

The findings pertaining to infection risks can also be interpreted as effects of the behavioural immune system. This theory proposes that, over the course of evolutionary history, humans developed a set of psychological mechanisms that provide informative cues that point to the potential presence of infectious pathogens and elicit relevant emotional and cognitive responses to avoid infection by the pathogens^10,11^. The behavioural immune system is an important defence mechanism against infectious pathogens but has some weaknesses. Because the system responds to an over-generalized set of superficial cues, the psychological mechanisms can inspire aversive reactions to things or people that pose no actual threat. Moreover, it is possible that the mechanisms do not respond to relevant cues that should indeed lead to pathogen avoidance and related behaviours, such as physical distancing.

A central concept of pathogen avoidance is disgust. In the course of human evolution, disgust evolved to detect and avoid stimuli that typically contain pathogens^12^. Its importance is underlined by the possibility of one-trial learning, e.g., after consuming mouldy food^13,14^. It plays an essential role in activating protective behaviour. For example, some obvious elicitors of disgust are body liquids or physical symptoms of infection, such as bruises, rash, coughing, and sneezing. Disgust is indeed examined as a mediator of the discussed effects of the behavioural immune system^9,10^. Although it has not been consistently identified as a mediator in the social identity model of risk taking^7^, disgust may play a more important role when infection risks are present than when we think of other health risks, such as alcohol consumption or bathing in frigid water. In fact, several studies support the assumption of lower feelings of disgust when we share a social identity. Mothers, for example, perceive their own baby’s faecal smell as less disgusting than that of someone else’s baby^15^. Likewise, university students rated a sweaty t-shirt with the logo of their own university as less disgusting than one with the logo of another university^16^. Thus, this source effect posits that the feelings of disgust elicited by a person will differ depending on one’s familiarity with that person: strangers will elicit more, familiar people less disgust^17^.

Disgust is understood as an important cue in the behavioural immune system theory. According to the theory, situations with an immediate threat of pathogens activate pathogen avoidance motivations^10,11^. Thus, situational pathogen avoidance includes affective, cognitive, and behavioural responses to social situations in which pathogen transmission is likely to occur^18^. Taking together the social identity model of risk taking^7^ and the behavioural immune system theory^10,11^, less situational pathogen avoidance should be activated when people interact with people with whom they share a social identity. As a consequence, they should feel less risk of infection and show fewer protective behaviours. This work takes a broad perspective on these social relations to explain variance in protective behaviour against COVID-19. Instead of assessing specific shared social identities (which could be manifold and vary by context), we assume that simply being with familiar others instead of strangers elicits a feeling of connectedness^19^, a state of psychological closeness that may eventually also lead to physical closeness. In regard to the behavioural immune system work, we focus on situational pathogen avoidance^18^ as an operating mechanism. Other factors can also affect COVID-19 protective behaviour^20^, e.g., the number of daily SARS-CoV-2 infections, attitudes toward the current regulations, and other risk factors, such as the vaccination status of another person. How strongly the feeling of connection to others who are present affects protective behaviour is an open question, given the plethora of other relevant factors that can indicate risk. The present studies therefore proposed to test the causal effect of familiarity with others on protective behaviours and perceived risk of infection and to explore connectedness and situational pathogen avoidance as mediators of that relationship. In addition, we aimed to learn the extent to which connectedness affects protective behaviours relative to other relevant cues and factors.

## Results

In two experiments conducted in Germany in October 2020 (n = 1022) and October 2021 (n = 994), we described a party scenario and varied the familiarity of the other guests. In Study 2, we additionally tested whether other cues of risk (e.g., receiving no information about the vaccination status of the other person) affected protective behaviours and the perceived risk of infection. In sum, the results provide supporting causal evidence for the relationship between higher familiarity and less perceived risk of infection as well as fewer COVID-19 protective behaviours, which was mediated by increased feelings of social connectedness and less situational pathogen avoidance. Study 2 indicates that connectedness had more influence on protective behaviours than other cues of risk, such as the COVID-19 risk status of the other person or one’s own COVID-19 vaccination status. Moreover, in a large-scale survey (Study 3, n = 23,023, October 2021–February 2022), participants were asked to recall their most recent physical encounter with others and state how connected they felt with those people and how much they had protected themselves during that encounter. The design allowed for estimating the relative effect of connectedness in a dynamic, real-life environment, controlling for other important aspects that may affect protective behaviours. The results suggest that the extent to which people felt connected to other people explained more variance in protective behaviours than the pandemic situation or attitudes toward protective behaviours. The following sections describe the studies in detail.

### Experimental results

In Study 1, the participants randomly read one of two scenarios, imagining themselves as either attending the birthday party of a friend who also invited other friends (familiarity scenario) or attending the birthday party of a friend who invited unknown people (stranger scenario). After the participants read the scenario, they indicated how connected they felt to the other guests, rated their risk of getting infected, and indicated the extent to which they would adopt behaviours to prevent COVID-19 in the given situation (three items on physical distancing, mask wearing, and ventilation of rooms that were averaged for analysis). Further questions measured the participants’ situational pathogen avoidance. All items were assessed on 7-point scales, with higher values indicating stronger perceptions or behaviours.

**Figure 1:**
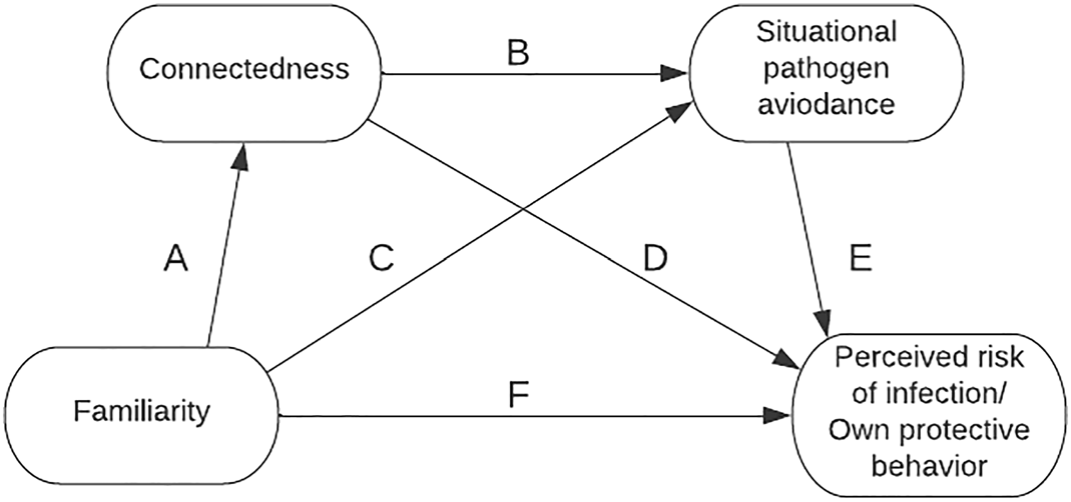
Multiple mediation model predicting perceived risk of infection and protective behaviours. Note: Data from the present studies show that greater familiarity was related to greater connectedness, which was associated with less situational pathogen avoidance. Less situational pathogen avoidance was related to lower perceived risks of infection and fewer protective behaviour. In all analyses the multiple mediation via connectedness and disgust were significant.

Figure 1, Tables 1, and 2 display the results of two serial mediation analyses that explored whether connectedness and situational pathogen avoidance mediated the effect of familiarity (friends vs. strangers) on the perceived risk of infection (model 1) and on protective behaviours (model 2). Indeed, greater familiarity was related to greater connectedness, which was in turn associated with less situational pathogen avoidance. Furthermore, the perceived risk of infection was significantly influenced by connectedness and situational pathogen avoidance, but there was no direct effect of familiarity. In sum, there was a significant indirect effect of familiarity on perceived risk, with connectedness and situational pathogen avoidance as mediators.

**Table 1:** Mediation analyses model 1.

Predictors	Dependent variable			Indirect effects	
	Perceived risk of infection	Connectedness	Situational pathogen avoidance	Estimate	Bias-corrected bootstrap 95% confidence interval
Familiarity	0.08 (0.47)	1.47 (< 0.001*)	0.18 (0.07)		
Connectedness	− 0.10 (< 0.001*)		− 0.22 (< 0.001*)		
Situational pathogen avoidance	0.38 (< 0.001*)				
Total				− 0.2	**[**− **0.32, **− 0.08]*
FA → CO → PR				− 0.15	**[**− **0.32, **− 0.08]*
FA → SPA → PR				0.07	[− 0.24, 0.14]
FA → CO → SPA → PR				− 0.12	**[**− **0.17, **− 0.08]*

In Study 1, two mediation models with two mediators (connectedness and disgust) were calculated. Mediation model 1 refers to the outcome variable perceived risk of infection. Table 1 shows the direct effects (β) in the mediation models and the indirect effects in mediation model 1. p-values less than 0.05 are assumed to be significant (*). An indirect effect is assumed as significant if the 95% CI does not include 0 (*).

**Table 2:** Mediation analyses model 2.

Predictors	Dependent variable			Indirect effects	
	Protective behaviour	Connectedness	Situational pathogen avoidance	Estimate	Bias-corrected bootstrap 95% confidence interval
Familiarity	0.15 (0.04*)	1.47 (< .001*)	0.18 (0.07)		
Connectedness	− 0.13 (< 0.001*)		− 0.22 (< 0.001*)		
Situational pathogen avoidance	0.51 (< 0.001*)				
Total				− 0.26	**[**− **0.38, **− 0.14]*
FA → CO → PB				− 0.19	**[**− **0.26, **− 0.12]*
FA → SPA → PB				0.09	[− 0.01, 0.19]
FA → CO → SPA → PB				− 0.16	**[**− **0.22, **− 0.12]*

In study 1, two mediation models with two mediators (connectedness and disgust) were calculated. Mediation model 2 refers to the outcome variable own protective behaviour. Table 2 shows the direct effects (β) in the mediation models and the indirect effects in mediation model 2. p-values less than 0.05 are assumed to be significant (*). An indirect effect is assumed as significant if the 95% CI does not include 0 (*).

When analysing protective behaviours as a dependent variable (model 2), the pattern of results was very similar. Greater familiarity was related to greater connectedness, which was associated with less situational pathogen avoidance, which was related to fewer protective behaviours. As in model 1, there was a significant indirect effect of familiarity on one’s own protective behaviours, with connectedness and situational pathogen avoidance as mediators. Contrary to model 1, there was also a significant direct effect of greater familiarity being related to more protective behaviours.

Additionally, we explored whether perceived risk of infection affects protective behaviour in the mediation model. We calculated another mediation model with connectedness, situational pathogen avoidance, and perceived risk of infection as mediators and protective behaviour as the outcome. This analysis showed a significant indirect effect of familiarity on protective behaviour via connectedness, situational pathogen avoidance and perceived risk of infection as mediators (for details, see the online supplement).

The second study aimed to (i) assess the effect of other risk factors, as the pandemic situation had changed considerably, and (ii) replicate the results of Study 1 as preregistered hypotheses. While no vaccine against COVID-19 had been approved during Study 1, around 70% of the German population had already received at least one dose of a COVID-19 vaccine during Study 2^21^. Moreover, at that time, rapid testing was being used to grant access to social events, e.g., to go to a concert or restaurant. Immunity to COVID-19 due to vaccination may have affected whether connectedness and situational pathogen avoidance still served as cues for potential infection risks. The same could be true for vaccinated others or those who had just received a negative rapid test result for COVID-19; because it could be assumed that the risk of transmitting was lower for vaccinated or tested others, being close to vaccinated and tested others may have activated the behavioural immune system to a lesser degree.

To test these assumptions, we adopted a 2 (familiarity: talking to a friend vs. a stranger) × 3 (other’s risk status: vaccinated vs. tested vs. no information) experimental design. In contrast to Study 1, familiarity with one person was manipulated by describing a conversational partner as either a friend or a stranger. The risk status of the conversational partner at the party was manipulated by providing information that the other person either (i) was vaccinated against COVID-19, (ii) had a negative rapid test result, or (iii) provided no information regarding COVID-19 risk status. After the participants had read one of the resulting six scenarios, they answered the same items as in Study 1 (connectedness, protective behaviours, perceived risk of infection, situational pathogen avoidance). The participants also indicated whether or not they were vaccinated against COVID-19.

As the prerequisites for the preregistered MANOVAs were not met, we present the analyses only in the supplement. The analyses used familiarity, risk status, and their interaction as predictors and found no effects of the experimental factors on perceived risk or behaviour (all *F*s < 1).

We further explored whether objective risk factors (the other person’s risk status and one’s own COVID-19 vaccination status) affected the reaction of the psychological immune system, i.e., situational pathogen avoidance. An analysis of variance with situational pathogen avoidance as a dependent variable and with familiarity, the other’s COVID-19 risk status, and one’s own vaccination status and their various interactions as predictors found no significant effect from familiarity, the other’s COVID-19 risk status, or one’s own COVID-19 vaccination status and revealed no significant interaction effects (for details, see the online supplement).

To replicate the findings of Study 1, the two serial mediation analyses depicted in Fig. 1 were conducted for perceived risk of infection (model 3, Table 3) and protective behaviours (model 4, Table 4). The data were collapsed across the three risk-status conditions. Greater familiarity was again related to greater connectedness, which was related to less situational pathogen avoidance. Less situational pathogen avoidance was related to less perceived risk of infection. Replicating the central result of Study 1, there was a significant indirect effect of greater connectedness and less situational pathogen avoidance mediating the relationship between familiarity and perceived risk of infection.

**Table 3:** Mediation analyses model 3.

Predictors	Dependent variable			Indirect effects	
	Perceived risk of infection	Connectedness	Situational pathogen avoidance	Estimate	Bias-corrected bootstrap 95% confidence interval
Familiarity	− 0.13 (0.23)	1.9 (< 0.001*)	0.07 (0.56)		
Connectedness	0.06 (0.05*)		− 0.17 (< 0.001*)		
Situational pathogen avoidance	0.34 (< 0.001*)				
Total				0.02	[− 0.12, 0.16]
FA → CO → PR				0.11	[− 0.02, 0.24]
FA → SPA → PR				0.02	[− 0.06, 0.11]
FA → CO → SPA → PR				− 0.11	**[**− **0.17, **− 0.06]*

In study 2, two mediation models with two mediators (connectedness and disgust) were calculated. Mediation model 3 refers to the outcome variable perceived risk of infection. Table 3 shows the direct effects (β) in the mediation models and the indirect effects in mediation model 3. p-values less than 0.05 are assumed to be significant (*). An indirect effect is assumed as significant if the 95% CI does not include 0 (*).

**Table 4:** Mediation analyses model 4.

Predictors	Dependent variable			Indirect effects	
	Protective behaviour	Connectedness	Situational pathogen avoidance	Estimate	Bias-corrected bootstrap 95% confidence interval
Familiarity	0.21 (0.03*)	1.9 (< 0.001*)	0.07 (0.56)		
Connectedness	− 0.08 (0.004*)		− 0.17 (< 0.001*)		
Situational pathogen avoidance	0.54 (< 0.001*)				
Total				− 0.29	**[**− **0.46, **− 0.14]*
FA → CO → PR				− 0.15	**[**− **0.27, **− 0.04]*
FA → SPA → PR				0.04	[− 0.1, 0.17]
FA → CO → SPA → PR				− 0.18	**[**− **0.26, **− 0.1]*

In study 2, two mediation models with two mediators (connectedness and disgust) were calculated. Mediation model 2 refers to the outcome variable own protective behaviour. Table 4 shows the direct effects (β) in the mediation models and the indirect effects in mediation model 4. p-values less than 0.05 are assumed to be significant (*). An indirect effect is assumed as significant if the 95% CI does not include 0 (*).

When the analysis was repeated with protective behaviour as the dependent variable (model 4), the main results were replicated as expected: greater familiarity was related to greater connectedness, which was accompanied by less situational pathogen avoidance. One’s own protective behaviour was significantly influenced by greater connectedness, less situational pathogen avoidance, and greater familiarity. Moreover, the mediation analysis showed a significant indirect effect of familiarity on one’s own protective behaviour, with connectedness and situational pathogen avoidance as mediators. We repeated the analyses for models 3 and 4 and controlled for the participants’ own vaccination status and estimated vaccination rate of the individual social group (for details, see the online supplement). The controlled analyses replicated the main findings of models 3 and 4. The preregistration had omitted connectedness, so, for the sake of comparability, we used the same models across both studies. The preregistered model with situational pathogen avoidance as a single mediator between familiarity and risk perceptions/behaviours yielded no significant effects. Familiarity is a binary variable, but connectedness has greater variability, which may partially explain the effect. Moreover, using connectedness as another mediator is logical, as it may be assumed that some people feel more connected to their friends than others. For that reason, we added connectedness as a second mediator in mediation models 1–4 (see Fig. 1).

As in Study 1, we explored the role of perceived risk of infection as another mediator in the model to predict protective behaviour as an outcome. The results again indicate a significant indirect effect from familiarity to protective behaviour via connectedness, situational pathogen avoidance, and perceived risk of infection as mediators (for details, see the online supplement).

### Survey on real-world behaviour

**Figure 2:**
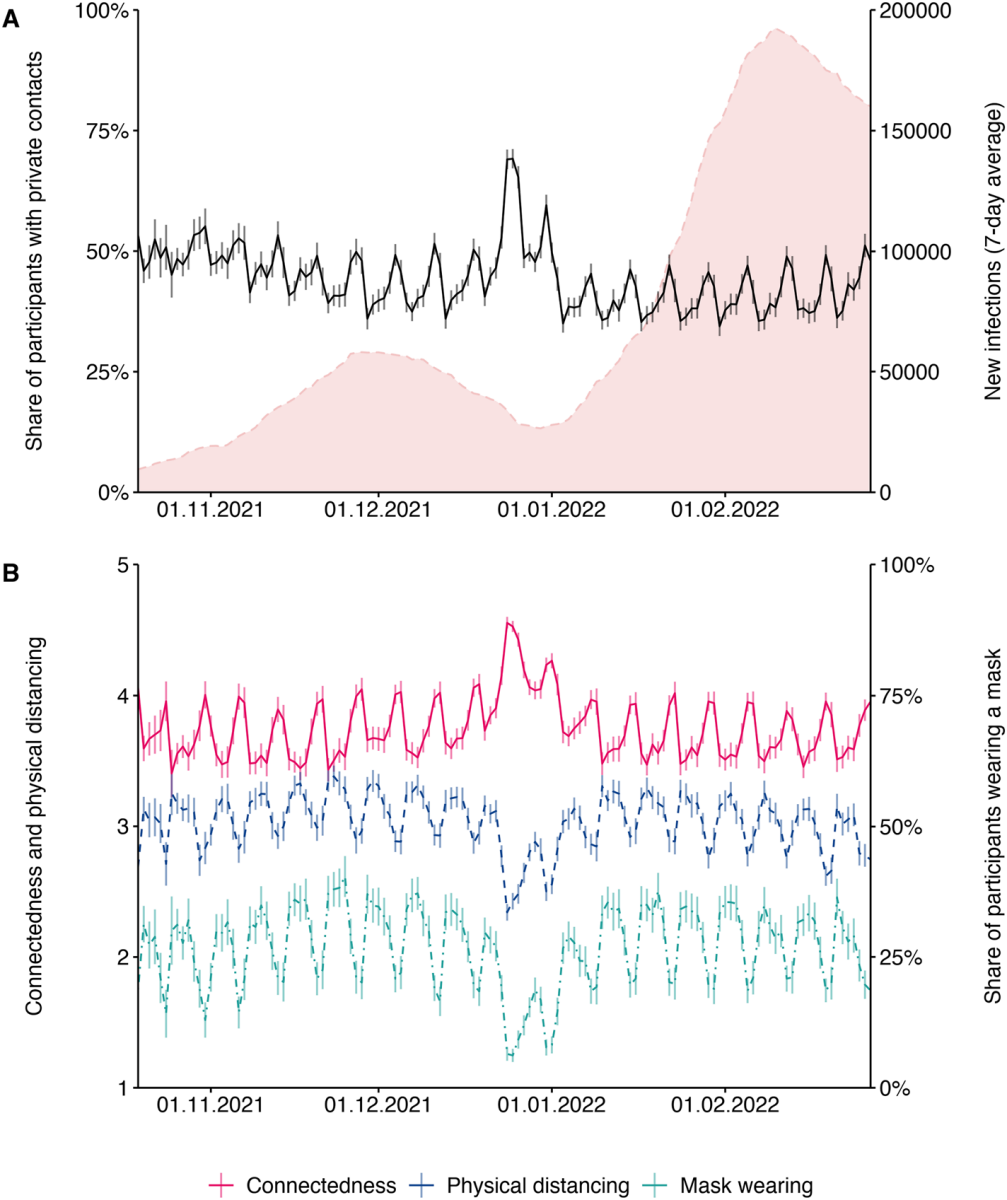
Covariation of connectedness towards others, physical distancing, and mask wearing. *Note*: (**A**) Between October 2021 and February 2022, the share of participants indicating to have private contacts outside their own household slightly decreased as the number of infections increased. (**B**) For participants who reported having had private contacts during the last day, perceived connectedness, and protective behaviours during the meetups (physical distancing and mask wearing) were negatively correlated. On weekends and over the holiday season at the end of 2021, private contacts were more likely, connectedness was higher, and protective behaviours were shown less than on weekdays and outside the holiday season. Translucent vertical bars indicate 95% confidence intervals. While connectedness and physical distancing were measures on a 5-point scale from “not at all” to “very much” (left y axis), mask wearing was assessed in a binary way (“yes” vs. “no”, right y-axis).

The results of Studies 1 and 2 were obtained in online experiments. While these are suitable for testing causal links, external validity may be low due to the fictitious and somewhat artificial study materials. Study 3 therefore assessed the assumed relations between connectedness, risk perception, and protective behaviours in actual, real-life behaviours. From October 21, 2021 through February 28, 2022, n = 22,777 people took part in a survey conducted in the context of the Corona Data Donation Project (Corona-Datenspende-App), a smartphone app that also collected biological data (e.g., sleep and activity data) via activity trackers. The participants were invited to take part in the survey via the app. In each survey, the participants were asked whether, on the previous day, they had privately met with other people who did not belong to their household. If so, the participants were further asked how connected they felt to those people, whether or not they wore a mask in the situation, and the extent to which they maintained physical distance from the other person. As may be seen in Fig. 2A, the number of such private gatherings slightly decreased after October 2021 (except during the Christmas holidays), indicating more cautious behaviour overall when infection rates increased. However, this change was small. It may be absent or weaker in the general population, given that our sample should be considered more compliant with pandemic regulations and recommendations than the rest of public. Over the holiday season, the number of private contacts increased while the perceived risk of getting infected decreased considerably, despite more contacts and less protective behaviour (Fig. 2B). In line with the results of the previously reported experiments, this was related to greater connectedness. Excluding the holiday season, the reported levels of connectedness to others and protective behaviours in private meetings barely changed over the course of the pandemic. Looking at weekly variations, connectedness with private contacts was higher on weekends than on weekdays, yet physical distancing and the share of participants wearing a mask were lower on weekends, indicating a negative relationship between connectedness and protective behaviours. Indeed, the Pearson-correlations with connectedness were negative for both physical distancing (*r* = − 0.39, *p* < 0.001) and mask wearing (*r* = − 0.47, *p* < 0.001).

Two linear mixed-model regressions were conducted to explore the relative importance of connectedness in driving risk perceptions and protective behaviours. In these analyses, physical distancing and mask wearing were predicted by the slope of new infections, participants’ age and gender (factors known to affect protective behaviour^22,23^), general attitude toward protective measures, and connectedness to the private contacts (fixed effects); they were further controlled for participation in multiple questionnaires (modelled as a random intercept). In both regressions, connectedness was by far the strongest predictor of protective behaviour (see Table 5). This finding was robust across individuals and did not change when excluding the holiday season from the analysis. In line with the previously presented experimental results, the finding indicates that the characteristics of social situations are more important in explaining variance in protective behaviours than the pandemic situation or attitudes toward those behaviours.

**Table 5:** Mixed model regressions from Study 3 predicting physical distancing and mask wearing.

Predictors	Physical distancing					Mask wearing				
	Estimates	Std. beta	CI	Std. CI	p	Estimates	Std. beta	CI	Std. CI	p
(Intercept)	43.86		42.03 to 45.68		**< 0.001**	6.21		5.66–6.77		**< 0.001**
Year of birth	− 0.02	− 0.20	− 0.02 to − 0.02	− 0.21 to − 0.19	**< 0.001**	− 0.00	− 0.08	− 0.00 to − 0.00	− 0.09 to − 0.07	**< 0.001**
Male (baseline: female)	− 0.08	− 0.03	− 0.11 to − 0.06	− 0.04 to − 0.02	**< 0.001**	− 0.01	− 0.01	− 0.02 to − 0.00	− 0.02 to − 0.01	**< 0.001**
New infections	0.00	0.01	0.00 to 0.00	0.01 to 0.02	**< 0.001**	0.00	0.01	0.00 to 0.00	0.00 to 0.01	**0.015**
Finding measures excessive	− 0.07	− 0.06	0.08 to − 0.06	− 0.06 to − 0.05	**< 0.001**	− 0.01	− 0.03	− 0.02 to − 0.01	− 0.04 to − 0.03	**< 0.001**
Connectedness	− 0.37	− 0.36	− 0.38 to − 0.37	− 0.36 to − 0.35	**< 0.001**	− 0.16	− 0.44	− 0.16 to − 0.16	− 0.45 to 0.44	**< 0.001**
Random effects										
σ^2^	0.76					0.11				
τ_00_	0.55_userId_					0.04_userId_				
ICC	0.42_userId_					0.26_userId_				
Observations	101,059					101,062				
Marginal R^2^/	0.189/					0.213/				
Conditional R^2^	0.530					0.417				

In Study 3, physical distancing and mask wearing behaviours in private contact situations were regressed on the slope of new infections, participants’ age and gender, their general attitude toward protective measures and their connectedness to the private contacts. Both analyses controlled for multiple participation (modelled as random intercept).

## Discussion

Individuals’ physical distancing is one of the more important behaviours to minimize the spread of the virus and reduce the number of infections in a global pandemic. While avoidance of contacts and gatherings was quite high at the beginning of the pandemic, physical distancing decreased over time, independent of the number of coronavirus cases in Germany^24^. Particularly, gatherings with friends and family caused an increasing number of cases^25^. One possible explanation is here provided by revealing the relationship between connectedness, situational pathogen avoidance, and behaviour. The mediation analyses in both experiments show that people feel greater connectedness to familiar people, such as friends. When connectedness is higher, people also feel less disgust as indicated by lower levels of situational pathogen avoidance. In turn, this is associated with lower risk perceptions and fewer protective behaviours, such as wearing a mask or ventilating rooms. Study 3 substantiates the experimental results and provides the same findings in real-life social interactions.

The findings support the social identity model of risk taking, which assumes that a shared social identity leads to lower risk perceptions and greater risk-taking behaviour^7,26^. We combined the model with assumptions from behavioural immune system theory and identified situational pathogen avoidance—a motivational tendency related to disgust—as a mechanism of the effect. While this needs testing in further studies, we cautiously conclude that, at least for infection risks, situational pathogen avoidance seems to be a relevant social cue. We suggest that its functionality can be blurred by social closeness, so efforts to effectively protect against the transmission of infectious diseases should take this effect into account. However, we also found limiting results, as situational pathogen avoidance was not affected by ostensibly “objective” risk factors, such as whether a person was vaccinated. However, we must concede the limitation that Study 2 was a fictitious questionnaire study only. The results may vary when people are confronted with vaccinated or unvaccinated people in the real world.

This research offers a plethora of behavioural insights from experimental and survey data, but it has several limitations. The second study was preregistered, but the analysis plan was adapted as detailed in the results. Instead of calculating a mediation with one mediator, a serial mediation was conducted, because connectedness proved to be an important component in the relationship. From this, we conclude that familiarity alone is not the relevant psychological basis of the effect, yet people indeed need to feel close to others for the behavioural immune system to disregard safety measures.

A further limitation of the experiments is the order of items. The participants first read the scenario and subsequently answered questions about connectedness (mediator), risk perception, protective behaviour (dependent variables), and situational pathogen avoidance (mediator). This was done to limit social desirability effects; thus, the actual effects may be underestimated. Future research could focus on causal relations, using our correlational evidence as a first indicator of important behavioural insights in health communication. Furthermore, the manipulation of COVID-19 risk status in Study 2 did not include the option that the conversation partner was neither vaccinated against COVID-19 nor had a negative test result. Future research should add this option in the manipulation to explore whether there is a difference between having no information about COVID-19 risk status and knowing that the other person is neither vaccinated nor tested. As the majority were vaccinated at the time of the study, the participants may have assumed that the other person belonged to the large group of vaccinated people (or had tested negative, as this was the requirement for attending large gatherings at the time of the study). Moreover, as we aimed to explain the effect of familiarity due to the behavioural immune system theory, the study did not evaluate other possible pathways like social norms which could also explain the effect of familiarity on protection behaviour^27^. In addition, further studies should investigate whether even higher effects can be found for gatherings with family members. Because family ties are usually even stronger than relationships with friends, we can assume that at gatherings with family members, protective behaviours could be even less likely compared to gatherings with friends or gatherings with strangers.

The obtained findings are relevant as they provide insights into how people behave in a threatening situation. The implications—beware of more lenient protective behaviours when you are with those who you like—may be especially important in elderly homes, during family gatherings, and at large gatherings, such as weddings and funerals—i.e., on every occasion when people come together and feel close to one another. It may thus be advisable to increase awareness of this phenomenon. As Fig. 2 shows, close contacts with little protection increase on weekends and during holiday seasons, so the advice seems warranted. Moreover, such psychological effects should be taken into account when modelling the future course of pandemics, especially during festive seasons. The fact that we are most biased when we most need to protect those we love like our friends and family is of utmost importance in health behaviour interventions. Our results are not only relevant in relation to the COVID-19 pandemic; the effects of familiarity on protective behaviour should be considered for all infectious diseases. For example, during influenza season or during holidays where familiar people meet each other like Christmas, a campaign referring to the link between familiarity and protective behaviour could convince people to be more careful when they meet their family and friends. This knowledge may also be important in future pandemics or outbreaks of new diseases.

## Materials and methods

Ethical approval for all the studies was provided by the University of Erfurt’s IRB (Studies 1 and 2: #20200302/#20200501; Study 3: #20220414). All studies were performed in accordance with “Guidelines to ensure good scientific practice” from the German Research Foundation. Informed consent was obtained from all participants. All questionnaires, data, analysis codes, and analysis outputs are available online (https://osf.io/ksjn7/).

### Study 1

The first study was conducted on 27–28 October 2020 as part of the COVID-19 Snapshot Monitoring (COSMO) project^28^. No vaccines against the coronavirus had been approved at that time.

#### Participants

N = 1022 participants took part in the study. The distribution of ages (18–74 years), genders, and federal state were representative of the German population (for details, see the online supplement). The participants were financially compensated by the data collection company at its usual rate. All individuals between 18 and 74 years of age who completed the survey were eligible for inclusion in the analyses.

#### Manipulation of familiarity

Within a randomized controlled trial, the participants were divided into two groups. At the beginning of the study, the two groups were presented with different scenarios of a party with various guests. Half the participants read a scenario of attending the birthday party of a friend who also invited other friends. The other half of the participants read the scenario of attending the birthday party of a friend who also invited strangers. Both situations were described as taking place in private rooms. After the participants read one scenario, they were asked the questions described below. The participants completed the items at their own pace. (All the questionnaires are available online).

#### Procedure and measures

After reading one of the two scenarios, the participants received the same items as follows.

Connectedness: To measure connectedness, the participants had to describe how connected they felt to the other guests at the party (*1* = *not connected at all* to *7* = *strongly connected*).

Perceived risk of infection: The participants rated their chance of getting infected at the party (*1* = *very low* to *7* = *very high*).

Protective behaviours: The questionnaire collected ratings on the extent to which the participants would observe recommended protective behaviours in the situation (*1* = *not at all* to *7* = *definitely*). The three items were keeping a distance from other people, ventilation of the rooms, and wearing a mask. The mean scores of the three variables were used for analysis (Cronbach’s alpha = 0.79, omega = 0.81).

Situational pathogen avoidance: Common disgust scales fail to map disgust as the broader experience of pathogen avoidance, which is the critical cue to activate the behavioural immune system^18^. According to behavioural immune system theory, situations with an immediate threat of pathogens activate pathogen avoidance motivations^10,11^. While simple measures of disgust do not cover broader cognitive and motivational aspects of pathogen avoidance, the Situational Pathogen Avoidance (SPA) scale captures the affective, cognitive, and behavioural responses to social situations in which pathogen transmission is likely to occur (with items such as “Right now, if I was standing next to a person who sneezed, I would feel disgusted” and “Right now, if someone coughed next to me without covering their mouth, I would move away from them”)^18^. Instead of focusing on disgust, therefore, we used the SPA scale, which represents a related but more comprehensive construct than disgust. Thus, the participants answered four items adapted from the SPA scale^18^. The mean scores of the four variables were used for analysis (Cronbach’s alpha = 0.85, omega = 0.89).

### Study 2

Study 2 was also part of the COSMO project. Data collection took place on 5–6 October 2021, at which time 70% of the German adult population was already vaccinated and rapid home testing was recommended for private gatherings.

#### Participants

N = 994 participants took part in the study. The sample characteristics and inclusion criteria mirror those of Study 1 (for details, see the online supplement).

#### Manipulation of familiarity and COVID-19 risk status

The experiment implemented a 2 (familiarity: friend vs. stranger) × 3 (COVID-19 risk status: vaccinated vs. not vaccinated but tested vs. no information) between-subject design. The participants were randomly assigned to one of the six resulting groups and had to imagine attending a birthday party where they had a conversation with either a friend or an unknown person (manipulating familiarity). Moreover, the participants received information that the other person was either vaccinated against Covid-19, was unvaccinated but had a negative rapid test result, or provided no information on risk status.

#### Procedure and measures

After the participants read one scenario, they were asked the same questions as in Study 1. In contrast to Study 1, however, the participants also indicated their connectedness to the person with whom they had a conversation. Moreover, the participants indicated whether they themselves were vaccinated against COVID-19 (yes/no) and estimated how many people in their circle of friends had been vaccinated (0.0–100%).

### Study 3

At the beginning of the COVID-19 pandemic, the Robert Koch Institute (Germany’s centre for disease control) released the Corona-Datenspende-App, a mobile application^29^ which enabled German residents age 16 or older with a smart watch or similar device to submit data on a voluntary basis. Initially, the data included only vital signs and sleep data. In mid-October 2021, a weekly survey module was added that asked users about their feelings, attitudes, and behaviours. This app was used for data collection in Study 3. The pandemic context (daily cases) is displayed in Fig. 2A. During the time of data collection, vaccination was widely available, and 70–77% of the German population were vaccinated. In the sample used, 99% were vaccinated, indicating that participants were more compliant with pandemic regulations and recommendations than the general population. At the time of the study, meeting people who did not belong to one’s household was restricted by various pandemic regulations, such as nighttime curfews and bans on large gatherings.

#### Participants

Between October 21, 2021, and February 28, 2022, n = 27,126 users completed the weekly survey at least once. In the analyses, however, only n = 22,777 participants were considered who indicated that, on the previous day, they had met in private with other people who did not belong to their household. In total, 101,741 completions were registered from these participants. Most of them (75%) were born between 1960 and 1990, and they included fewer females (63% male) than the general German population (for details, see the online supplement).

#### Measures

Connectedness: The participants were asked how connected they felt to the ones they had met. The answers were recorded on a 5-point scale ranging from “not at all” to “very much”.

Protective behaviours: The participants were asked how much physical distance they had kept from the others (recorded on a 5-point scale ranging from “not at all” to “very much”) and whether they had worn a mask in the situation (“yes” vs. “no”).

## Supplementary Information

Supplementary Information 1.

Supplementary Information 2.

Supplementary Information 3.

Supplementary Information 4.

Supplementary Information 5.

Supplementary Information 6.

## Data Availability

All questionnaires, data, analysis codes, and analysis outputs for study 1 and study 2 are available online (https://osf.io/ksjn7/). The data for study 3 are available on request from Marc Wiedermann.
