# Supplementary material for: Situational pathogen avoidance mediates the impact of social connectedness on preventive measures during the COVID-19 pandemic
Source: Sci Rep. 2023 Feb 10;13:2418. doi: 10.1038/s41598-023-29239-y (PMC9912235; doi:10.1038/s41598-023-29239-y)
Supplement: Supplementary file 4 — Supplementary Information 4. [file 41598_2023_29239_MOESM4_ESM.docx]

Procedure study 2

Die Teilnehmenden wurden randomisiert zu einer der sechs unten beschriebenen Gruppen zugeteilt. Es wurden die gleichen Fragen gestellt.

The participants are randomly assigned to one of the six groups described below. They were asked the same questions.

|  | Gruppe 1  (Freund + geimpft)  Group 1 (Friend + vaccinated) | Gruppe 2  (Freund + getestet)  Group 2 (Friend + tested) | Gruppe 3  (Freund + keine Information)  Group 3 (Friend + no information) | Gruppe 4 (Unbekannte Person + geimpft)  Group 4 (Unknown person + vaccinated) | Gruppe 5 (Unbekannte Person + getestet)  Group 5 (Unknown person + tested) | Gruppe 6  (Unbekannte Person + keine Information)  Group 6 (Unknown person + no information) |
| --- | --- | --- | --- | --- | --- | --- |
| Manipulation | Sie sind auf einer Geburtstagsfeier. Der Gastgeber hat neben Ihnen viele Ihrer Freunde eingeladen. Die Feier findet in privaten Räumen statt. Auf der Feier unterhalten Sie sich mit einer Person, die Sie gut kennen. Sie erfahren, dass diese Person geimpft ist.  You are at a birthday party. The host invited many of your friends in addition to you. The party takes place in private rooms. At the party you talk to a person you know well. You learn that this person is vaccinated. | Sie sind auf einer Geburtstagsfeier. Der Gastgeber hat neben Ihnen viele Ihrer Freunde eingeladen. Die Feier findet in privaten Räumen statt. Auf der Feier unterhalten Sie sich mit einer Person, die Sie gut kennen. Sie erfahren, dass diese Person nicht geimpft, aber getestet ist.  You are at a birthday party. The host invited many of your friends in addition to you. The party takes place in private rooms. At the party you talk to a person you know well. You learn that this person is not vaccinated but tested. | Sie sind auf einer Geburtstagsfeier. Der Gastgeber hat neben Ihnen viele Ihrer Freunde eingeladen. Die Feier findet in privaten Räumen statt. Auf der Feier unterhalten Sie sich mit einer Person, die Sie gut kennen.  You are at a birthday party. The host invited many of your friends in addition to you. The party takes place in private rooms. At the party you talk to a person you know well. | Sie sind auf einer Geburtstagsfeier. Der Gastgeber hat neben Ihnen nur Ihnen unbekannte Menschen eingeladen. Die Feier findet in privaten Räumen statt. Auf der Feier unterhalten Sie sich mit einer Person, die Sie gerade auf der Feier kennengelernt haben. Sie erfahren, dass diese Person geimpft ist.  You are at a birthday party. The host invited many of your friends in addition to you. The party takes place in private rooms. At the party you talk to a person that you just met at the party. You learn that this person is vaccinated. | Sie sind auf einer Geburtstagsfeier. Der Gastgeber hat neben Ihnen nur Ihnen unbekannte Menschen eingeladen. Die Feier findet in privaten Räumen statt. Auf der Feier unterhalten Sie sich mit einer Person, die Sie gerade auf der Feier kennengelernt haben. Sie erfahren, dass diese Person nicht geimpft, aber getestet ist.  You are at a birthday party. The host invited many of your friends in addition to you. The party takes place in private rooms. At the party you talk to a person that you just met at the party. You learn that this person is not vaccinated but tested. | Sie sind auf einer Geburtstagsfeier. Der Gastgeber hat neben Ihnen nur Ihnen unbekannte Menschen eingeladen. Die Feier findet in privaten Räumen statt. Im Laufe der Feier unterhalten Sie sich mit einer Person, die Sie gerade kennengelernt haben.  You are at a birthday party. The host has invited only people unknown for you in addition to you. The party takes place in private rooms. At the party you talk to a person that you just met at the party. |
| Connectedness  CONNETCTED | Wie stark fühlen Sie sich mit den Gästen der Feier verbunden?  How strongly do you feel connected to the guests at the party?  1 gar nicht verbunden – 7 stark verbunden  1 not connected at all – 7 strongly connected | | | | | |
| Behavior  OWN_SPACE  OWN_AIR  OWN_MASK | Welche Vorsichtsmaßnahmen wenden Sie an, damit das Risiko einer Ansteckung auf der Feier minimiert wird?  Which protective measures do you use to minimize the risk of infection at the party?  – Abstand von 1,5m zu anderen Menschen einhalten  – keep the distance of 1,5m to other people  – Geschlossene Räume regelmäßig lüften  – regularly ventilate closed rooms  – Maske tragen  – Wear a mask  1 Nein, wende ich auf keinen Fall an – 7 Ja, wende ich auf jeden Fall an  1 No, I don’t use in any case – 7 Yes, I use in any case | | | | | |
| Risk  RISK | Wie hoch schätzen Sie die Wahrscheinlichkeit ein, dass Sie sich in der beschriebenen Situation mit dem Coronavirus infizieren werden?  How likely do you think you are to become infected with coronavirus in the situation described?  1 sehr gering – 7 sehr hoch  1 very low – 7 very high | | | | | |
| Situational Pathogen Avoidance  SPA_SNEEZE  SPA_  HANDSHAKE  SPA_COUGH  SPA_SPACE | Wie sehr stimmen Sie den folgenden Aussagen zu?  How much do you agree with the following statements?  Wenn ich neben einer Person stünde, die niest, würde ich mich angewidert fühlen.  If I were standing next to a person who sneezed, I would feel disgusted  Wenn ich die Hand der Anderen schütteln würde, wäre ich angewidert.  If I were shaking the hand of others I would feel disgusted.  Wenn jemand neben mir husten würde, ohne seinen Mund zu bedecken, würde ich mich von ihm wegbewegen.  If someone next to me coughed without covering his mouth I would move away from him.  Wenn die Anderen nicht genügend Abstand hielten, würde ich mich bedroht fühlen.  If the others did not keep enough distance, I would feel threatened.  1 Stimme überhaupt nicht zu – 7 Stimme voll und ganz zu  1 do not agree at all – 7 fully agree | | | | | |
| Vaccinated people  ESTIMATED_  VACCINATED | Bitte schätzen Sie, wie viel Prozent der Personen aus Ihrem nahen Umfeld bereits vollständig gegen das Coronavirus geimpft  sind.  Please estimate what percentage of people in your close environment have already been fully vaccinated against the coronavirus. | | | | | |

Demographical variables were collected before the experiment

| Variable | Item | Answers |
| --- | --- | --- |
| AGE | Wie alt sind Sie?  How old are you? | Alter in Jahren  Age in years |
| GENDER | Welches Geschlecht haben Sie?  What gender are you? | 1 männlich  2 weiblich  1 male  2 female |
| EDUCATION | Bitte machen Sie Angaben zu Ihrer Schulbildung  Please provide information about your school education | 1 Bis zu 9 Jahre Schulbildung  2 Mindestens 10 Jahre Schulbildung (ohne Hochschulreife)  3 Mindestens 10 Jahre Schulbildung (mit Hochschulreife)  1 up to 9 years of school education  2 at least 10 years of school education (without University entrance qualification)  3 at least 10 years of school education (with University entrance qualification) |
