# Supplementary material for: Situational pathogen avoidance mediates the impact of social connectedness on preventive measures during the COVID-19 pandemic
Source: Sci Rep. 2023 Feb 10;13:2418. doi: 10.1038/s41598-023-29239-y (PMC9912235; doi:10.1038/s41598-023-29239-y)
Supplement: Supplementary file 6 — Supplementary Information 6. [file 41598_2023_29239_MOESM6_ESM.html]

We are having a party


# We are having a party

#### Frederike Taubert

#### 08/08/2022

# Materials and Methods

## Questionnaire and recruitment

The experiments were part of a 15- to 20-minute online questionnaire with a serial cross-sectional design and multiple data collection components. For study 1, data were collected between 27. – 28.10.2020. For study 2, data were collected between 5. – 6.10.2021. At each data collection, we aimed to collect 1,000 complete data sets. Participants were recruited via an external study sample provider (www.respondi.de), certified according to ISO 26362. Participants took part in the survey voluntarily and received renumeration paid by the data collection company. Participants were German-speaking respondents living in Germany, and the quota sample matched current distributions in terms of age x gender (crossed) and residency in a German federal state (not crossed). Data collection took place online. For more details, questionnaires, and codebooks, see OSF https://osf.io/ksjn7/.

## Experimental manipulation

In both experimental studies, participants should introduce themselves to visit a birthday party from a friend. In study 1, the familiarity to the other guest were manipulated between two groups. On half of the participants read the scenario of a birthday party from a friend who also invited other friends, while the other half of participants read the scenario of a party from a friend who also invited unknown people. Study 2 included a 2 (familiarity: friend vs. stranger) x 3 (COVID-19 risk status: vaccinated vs. not vaccinated but tested vs. no information) between subject design. Participants were randomly distributed to one of the six resulting groups. Within the scenario, participants had to imagine visiting a birthday party where they had a conversation either with a friend or an unknown person, manipulating familiarity. Moreover, participants received the information that the other person was either vaccinated against Covid-19, was unvaccinated but had a negative rapid test result. In the third condition participants received no information on the risk status. The full texts for all manipulations are avoidable on OSF https://osf.io/ksjn7/.

### Variables

1. Connectedness (CONNECTED): To measure connectedness participants had to answer how much they feel connected to the other guests at the party (1 not connected at all – 7 strongly connected).
2. Situational pathogen avoidance (SPAx): Participants were asked about their situational pathogen avoidance (4 items, 1 do not agree at all – 7 fully agree). The items were adapted from the Situational Pathogen Avoidance Scale (22). For analyses, the mean score of the four variables was used.

```
## Omega 
## Call: omegah(m = m, nfactors = nfactors, fm = fm, key = key, flip = flip, 
##     digits = digits, title = title, sl = sl, labels = labels, 
##     plot = plot, n.obs = n.obs, rotate = rotate, Phi = Phi, option = option, 
##     covar = covar)
## Alpha:                 0.85 
## G.6:                   0.82 
## Omega Hierarchical:    0.82 
## Omega H asymptotic:    0.92 
## Omega Total            0.89 
## 
## Schmid Leiman Factor loadings greater than  0.2 
##                  g   F1*  F2*  F3*   h2   u2   p2
## SPA_SNEEZE    0.72       0.43      0.73 0.27 0.72
## SPA_HANDSHAKE 0.74 -0.25 0.36      0.74 0.26 0.75
## SPA_COUGH     0.70  0.28           0.57 0.43 0.86
## SPA_SPACE     0.83                 0.71 0.29 0.98
## 
## With eigenvalues of:
##    g  F1*  F2*  F3* 
## 2.26 0.17 0.32 0.00 
## 
## general/max  7.15   max/min =   72.76
## mean percent general =  0.83    with sd =  0.12 and cv of  0.14 
## Explained Common Variance of the general factor =  0.82 
## 
## The degrees of freedom are -3  and the fit is  0 
## The number of observations was  1022  with Chi Square =  0  with prob <  NA
## The root mean square of the residuals is  0 
## The df corrected root mean square of the residuals is  NA
## 
## Compare this with the adequacy of just a general factor and no group factors
## The degrees of freedom for just the general factor are 2  and the fit is  0.09 
## The number of observations was  1022  with Chi Square =  91.7  with prob <  1.2e-20
## The root mean square of the residuals is  0.06 
## The df corrected root mean square of the residuals is  0.1 
## 
## RMSEA index =  0.209  and the 10 % confidence intervals are  0.174 0.247
## BIC =  77.84 
## 
## Measures of factor score adequacy             
##                                                  g   F1*   F2*   F3*
## Correlation of scores with factors            0.92  0.59  0.61  0.07
## Multiple R square of scores with factors      0.84  0.34  0.37  0.01
## Minimum correlation of factor score estimates 0.68 -0.31 -0.26 -0.99
## 
##  Total, General and Subset omega for each subset
##                                                  g  F1*  F2* F3*
## Omega total for total scores and subscales    0.89 0.77 0.84  NA
## Omega general for total scores and subscales  0.82 0.76 0.65  NA
## Omega group for total scores and subscales    0.06 0.01 0.19  NA
```

```
## Omega 
## Call: omegah(m = m, nfactors = nfactors, fm = fm, key = key, flip = flip, 
##     digits = digits, title = title, sl = sl, labels = labels, 
##     plot = plot, n.obs = n.obs, rotate = rotate, Phi = Phi, option = option, 
##     covar = covar)
## Alpha:                 0.86 
## G.6:                   0.82 
## Omega Hierarchical:    0.83 
## Omega H asymptotic:    0.95 
## Omega Total            0.88 
## 
## Schmid Leiman Factor loadings greater than  0.2 
##                  g  F1*  F2*  F3*   h2   u2   p2
## SPA_SNEEZE    0.77      0.36      0.73 0.27 0.82
## SPA_HANDSHAKE 0.80                0.65 0.35 0.99
## SPA_COUGH     0.66      0.33      0.55 0.45 0.78
## SPA_SPACE     0.82                0.67 0.33 1.00
## 
## With eigenvalues of:
##    g  F1*  F2*  F3* 
## 2.35 0.00 0.24 0.02 
## 
## general/max  9.75   max/min =   Inf
## mean percent general =  0.9    with sd =  0.11 and cv of  0.13 
## Explained Common Variance of the general factor =  0.9 
## 
## The degrees of freedom are -3  and the fit is  0 
## The number of observations was  994  with Chi Square =  0  with prob <  NA
## The root mean square of the residuals is  0 
## The df corrected root mean square of the residuals is  NA
## 
## Compare this with the adequacy of just a general factor and no group factors
## The degrees of freedom for just the general factor are 2  and the fit is  0.05 
## The number of observations was  994  with Chi Square =  45.87  with prob <  1.1e-10
## The root mean square of the residuals is  0.05 
## The df corrected root mean square of the residuals is  0.08 
## 
## RMSEA index =  0.149  and the 10 % confidence intervals are  0.113 0.187
## BIC =  32.06 
## 
## Measures of factor score adequacy             
##                                                  g F1*   F2*   F3*
## Correlation of scores with factors            0.92   0  0.56  0.21
## Multiple R square of scores with factors      0.85   0  0.31  0.04
## Minimum correlation of factor score estimates 0.70  -1 -0.38 -0.91
## 
##  Total, General and Subset omega for each subset
##                                                  g F1*  F2* F3*
## Omega total for total scores and subscales    0.88  NA 0.78 0.8
## Omega general for total scores and subscales  0.83  NA 0.63 0.8
## Omega group for total scores and subscales    0.04  NA 0.15 0.0
```

3. Perceived risk of infection (RISK): Participants had to rate their chance of getting infected at the party (1 item, 1 very low – 7 very high).
4. Protective behavior (BEHAVIOR):The questionnaire collected ratings on the extent to which participants would show recommended protective behaviors in the situation (1 not at all – 7 definitely). The three items were keeping a distance from other people, ventilation of the rooms and wearing a mask. For analyses, the mean score of the three variables was used.

```
## Omega 
## Call: omegah(m = m, nfactors = nfactors, fm = fm, key = key, flip = flip, 
##     digits = digits, title = title, sl = sl, labels = labels, 
##     plot = plot, n.obs = n.obs, rotate = rotate, Phi = Phi, option = option, 
##     covar = covar)
## Alpha:                 0.79 
## G.6:                   0.74 
## Omega Hierarchical:    0 
## Omega H asymptotic:    0 
## Omega Total            0.81 
## 
## Schmid Leiman Factor loadings greater than  0.2 
##               g  F1*   F2*   F3*   h2   u2   p2
## OWN_SPACE       0.91             0.83 0.17 0.00
## OWN_AIR         0.63             0.42 0.58 0.01
## OWN_MASK        0.73             0.56 0.44 0.00
## 
## With eigenvalues of:
##    g  F1*  F2*  F3* 
## 0.01 1.75 0.05 0.00 
## 
## general/max  0   max/min =   Inf
## mean percent general =  0    with sd =  0.01 and cv of  1.73 
## Explained Common Variance of the general factor =  0 
## 
## The degrees of freedom are -3  and the fit is  0 
## The number of observations was  1022  with Chi Square =  0  with prob <  NA
## The root mean square of the residuals is  0 
## The df corrected root mean square of the residuals is  NA
## 
## Compare this with the adequacy of just a general factor and no group factors
## The degrees of freedom for just the general factor are 0  and the fit is  0.98 
## The number of observations was  1022  with Chi Square =  998.05  with prob <  NA
## The root mean square of the residuals is  0.56 
## The df corrected root mean square of the residuals is  NA 
## 
## Measures of factor score adequacy             
##                                                   g  F1*   F2* F3*
## Correlation of scores with factors             0.06 0.93  0.30   0
## Multiple R square of scores with factors       0.00 0.87  0.09   0
## Minimum correlation of factor score estimates -0.99 0.74 -0.82  -1
## 
##  Total, General and Subset omega for each subset
##                                                  g  F1* F2* F3*
## Omega total for total scores and subscales    0.81 0.81  NA  NA
## Omega general for total scores and subscales  0.00 0.00  NA  NA
## Omega group for total scores and subscales    0.81 0.81  NA  NA
```

```
## Omega 
## Call: omegah(m = m, nfactors = nfactors, fm = fm, key = key, flip = flip, 
##     digits = digits, title = title, sl = sl, labels = labels, 
##     plot = plot, n.obs = n.obs, rotate = rotate, Phi = Phi, option = option, 
##     covar = covar)
## Alpha:                 0.76 
## G.6:                   0.71 
## Omega Hierarchical:    0 
## Omega H asymptotic:    0 
## Omega Total            0.8 
## 
## Schmid Leiman Factor loadings greater than  0.2 
##               g  F1*   F2*   F3*   h2   u2 p2
## OWN_SPACE       0.94             0.88 0.12  0
## OWN_AIR         0.57  0.20       0.37 0.63  0
## OWN_MASK        0.70             0.52 0.48  0
## 
## With eigenvalues of:
##    g  F1*  F2*  F3* 
## 0.00 1.69 0.07 0.00 
## 
## general/max  0   max/min =   Inf
## mean percent general =  0    with sd =  0 and cv of  NaN 
## Explained Common Variance of the general factor =  0 
## 
## The degrees of freedom are -3  and the fit is  0 
## The number of observations was  994  with Chi Square =  0  with prob <  NA
## The root mean square of the residuals is  0 
## The df corrected root mean square of the residuals is  NA
## 
## Compare this with the adequacy of just a general factor and no group factors
## The degrees of freedom for just the general factor are 0  and the fit is  0.9 
## The number of observations was  994  with Chi Square =  889.12  with prob <  NA
## The root mean square of the residuals is  0.53 
## The df corrected root mean square of the residuals is  NA 
## 
## Measures of factor score adequacy             
##                                                   g  F1*   F2* F3*
## Correlation of scores with factors             0.03 0.95  0.35   0
## Multiple R square of scores with factors       0.00 0.90  0.12   0
## Minimum correlation of factor score estimates -1.00 0.79 -0.76  -1
## 
##  Total, General and Subset omega for each subset
##                                                 g F1* F2* F3*
## Omega total for total scores and subscales    0.8 0.8  NA  NA
## Omega general for total scores and subscales  0.0 0.0  NA  NA
## Omega group for total scores and subscales    0.8 0.8  NA  NA
```

# Sample characteristics

## Study 1

### Age

```
## 
## 18-29 30-49 50-64 65-74 
##   205   369   273   175
```

### Gender

```
## 
## female   male 
##    522    500
```

### Education

```
## 
##    10+ years (with university entrance diploma) 
##                                             597 
## 10+ years (without university entrance diploma) 
##                                             317 
##                                   up to 9 years 
##                                             108
```

### Dependent variable perceived risk of infection strafied by manipulation

### Dependent variable protective behavior strafied by manipulation

## Study 2

### Age

```
## 
## 18-29 29-50 50-64 65-74 
##   198   378   261   157
```

### Gender

```
## 
## female   male 
##    500    494
```

### Education

```
## 
##                                    10+ years 
##                                          309 
## 10+ years (with university entrance diploma) 
##                                          565 
##                                up to 9 years 
##                                          120
```

### Dependent variable perceived risk of infection strafied by manipulation

### Dependent variable protective behavior strafied by manipulation

# Results

## Study 1

### Mediation Model 1

Analysis:  
- Serial Mediation Model with PROCESS (Model 6)  
- DV: Perceived risk of infection  
- IV: Familiarity  
- Mediators: Connectedness, Situational pathogen avoidance

```
## 
## ********************* PROCESS for R Version 4.0.1 ********************* 
##  
##            Written by Andrew F. Hayes, Ph.D.  www.afhayes.com              
##    Documentation available in Hayes (2022). www.guilford.com/p/hayes3   
##  
## *********************************************************************** 
##                    
## Model : 6          
##     Y : RISK       
##     X : FAMILIARITY
##    M1 : CONNECTED  
##    M2 : SPAx       
## 
## Sample size: 1022
## 
## Random seed: 622470
## 
## 
## *********************************************************************** 
## Outcome Variable: CONNECTED
## 
## Model Summary: 
##           R      R-sq       MSE         F       df1       df2         p
##      0.3968    0.1574    2.9015  190.5758    1.0000 1020.0000    0.0000
## 
## Model: 
##                 coeff        se         t         p      LLCI      ULCI
## constant       0.9230    0.1686    5.4747    0.0000    0.5922    1.2538
## FAMILIARITY    1.4711    0.1066   13.8049    0.0000    1.2620    1.6802
## 
## Standardized coefficients:
##                 coeff
## FAMILIARITY    0.7931
## 
## *********************************************************************** 
## Outcome Variable: SPAx
## 
## Model Summary: 
##           R      R-sq       MSE         F       df1       df2         p
##      0.2543    0.0646    2.0879   35.2128    2.0000 1019.0000    0.0000
## 
## Model: 
##                 coeff        se         t         p      LLCI      ULCI
## constant       5.6345    0.1451   38.8311    0.0000    5.3497    5.9192
## FAMILIARITY    0.1788    0.0985    1.8155    0.0697   -0.0145    0.3720
## CONNECTED     -0.2189    0.0266   -8.2411    0.0000   -0.2710   -0.1668
## 
## Standardized coefficients:
##                 coeff
## FAMILIARITY    0.1198
## CONNECTED     -0.2720
## 
## *********************************************************************** 
## Outcome Variable: RISK
## 
## Model Summary: 
##           R      R-sq       MSE         F       df1       df2         p
##      0.3791    0.1437    2.4256   56.9451    3.0000 1018.0000    0.0000
## 
## Model: 
##                 coeff        se         t         p      LLCI      ULCI
## constant       2.3553    0.2463    9.5636    0.0000    1.8721    2.8386
## FAMILIARITY    0.0763    0.1063    0.7181    0.4729   -0.1323    0.2850
## CONNECTED     -0.0989    0.0296   -3.3435    0.0009   -0.1569   -0.0408
## SPAx           0.3828    0.0338   11.3365    0.0000    0.3165    0.4490
## 
## Standardized coefficients:
##                 coeff
## FAMILIARITY    0.0454
## CONNECTED     -0.1092
## SPAx           0.3400
## 
## ************************ TOTAL EFFECT MODEL *************************** 
## Outcome Variable: RISK
## 
## Model Summary: 
##           R      R-sq       MSE         F       df1       df2         p
##      0.0369    0.0014    2.8232    1.3894    1.0000 1020.0000    0.2388
## 
## Model: 
##                 coeff        se         t         p      LLCI      ULCI
## constant       4.3435    0.1663   26.1178    0.0000    4.0172    4.6699
## FAMILIARITY   -0.1239    0.1051   -1.1787    0.2388   -0.3302    0.0824
## 
## Standardized coefficients:
##                 coeff
## FAMILIARITY   -0.0737
## 
## ************ TOTAL, DIRECT, AND INDIRECT EFFECTS OF X ON Y ************
## 
## Total effect of X on Y:
##      effect        se         t         p      LLCI      ULCI      c_ps
##     -0.1239    0.1051   -1.1787    0.2388   -0.3302    0.0824   -0.0737
## 
## Direct effect of X on Y:
##      effect        se         t         p      LLCI      ULCI     c'_ps
##      0.0763    0.1063    0.7181    0.4729   -0.1323    0.2850    0.0454
## 
## Indirect effect(s) of X on Y:
##          Effect    BootSE  BootLLCI  BootULCI
## TOTAL   -0.2003    0.0621   -0.3234   -0.0783
## Ind1    -0.1454    0.0499   -0.2464   -0.0485
## Ind2     0.0684    0.0399   -0.0088    0.1482
## Ind3    -0.1233    0.0216   -0.1682   -0.0832
## 
## Partially standardized indirect effect(s) of X on Y:
##          Effect    BootSE  BootLLCI  BootULCI
## TOTAL   -0.1192    0.0370   -0.1922   -0.0464
## Ind1    -0.0865    0.0297   -0.1469   -0.0289
## Ind2     0.0407    0.0236   -0.0053    0.0874
## Ind3    -0.0733    0.0127   -0.0998   -0.0501
## 
## Indirect effect key:
## Ind1 FAMILIARITY    ->    CONNECTED    ->    RISK              
## Ind2 FAMILIARITY    ->    SPAx    ->    RISK              
## Ind3 FAMILIARITY    ->    CONNECTED    ->    SPAx    ->    RISK
## 
## ******************** ANALYSIS NOTES AND ERRORS ************************ 
## 
## Level of confidence for all confidence intervals in output: 95
## 
## Number of bootstraps for percentile bootstrap confidence intervals: 5000
## 
## NOTE: Standardized coefficients for dichotomous or multicategorical X are
##       in partially standardized form.
```

### Mediation Model 2

Analysis:  
- Serial Mediation Model with PROCESS (Model 6)  
- DV: Protective behaviors  
- IV: Familiarity  
- Mediators: Connectedness, Situational pathogen avoidance

```
## 
## ********************* PROCESS for R Version 4.0.1 ********************* 
##  
##            Written by Andrew F. Hayes, Ph.D.  www.afhayes.com              
##    Documentation available in Hayes (2022). www.guilford.com/p/hayes3   
##  
## *********************************************************************** 
##                    
## Model : 6          
##     Y : BEHAVIOR   
##     X : FAMILIARITY
##    M1 : CONNECTED  
##    M2 : SPAx       
## 
## Sample size: 1022
## 
## Random seed: 180672
## 
## 
## *********************************************************************** 
## Outcome Variable: CONNECTED
## 
## Model Summary: 
##           R      R-sq       MSE         F       df1       df2         p
##      0.3968    0.1574    2.9015  190.5758    1.0000 1020.0000    0.0000
## 
## Model: 
##                 coeff        se         t         p      LLCI      ULCI
## constant       0.9230    0.1686    5.4747    0.0000    0.5922    1.2538
## FAMILIARITY    1.4711    0.1066   13.8049    0.0000    1.2620    1.6802
## 
## Standardized coefficients:
##                 coeff
## FAMILIARITY    0.7931
## 
## *********************************************************************** 
## Outcome Variable: SPAx
## 
## Model Summary: 
##           R      R-sq       MSE         F       df1       df2         p
##      0.2543    0.0646    2.0879   35.2128    2.0000 1019.0000    0.0000
## 
## Model: 
##                 coeff        se         t         p      LLCI      ULCI
## constant       5.6345    0.1451   38.8311    0.0000    5.3497    5.9192
## FAMILIARITY    0.1788    0.0985    1.8155    0.0697   -0.0145    0.3720
## CONNECTED     -0.2189    0.0266   -8.2411    0.0000   -0.2710   -0.1668
## 
## Standardized coefficients:
##                 coeff
## FAMILIARITY    0.1198
## CONNECTED     -0.2720
## 
## *********************************************************************** 
## Outcome Variable: BEHAVIOR
## 
## Model Summary: 
##           R      R-sq       MSE         F       df1       df2         p
##      0.6121    0.3746    1.1833  203.2730    3.0000 1018.0000    0.0000
## 
## Model: 
##                 coeff        se         t         p      LLCI      ULCI
## constant       3.3388    0.1720   19.4098    0.0000    3.0012    3.6763
## FAMILIARITY    0.1540    0.0743    2.0735    0.0384    0.0083    0.2997
## CONNECTED     -0.1283    0.0207   -6.2147    0.0000   -0.1689   -0.0878
## SPAx           0.5079    0.0236   21.5369    0.0000    0.4616    0.5542
## 
## Standardized coefficients:
##                 coeff
## FAMILIARITY    0.1121
## CONNECTED     -0.1733
## SPAx           0.5519
## 
## ************************ TOTAL EFFECT MODEL *************************** 
## Outcome Variable: BEHAVIOR
## 
## Model Summary: 
##           R      R-sq       MSE         F       df1       df2         p
##      0.0392    0.0015    1.8855    1.5681    1.0000 1020.0000    0.2108
## 
## Model: 
##                 coeff        se         t         p      LLCI      ULCI
## constant       5.9795    0.1359   43.9968    0.0000    5.7128    6.2462
## FAMILIARITY   -0.1076    0.0859   -1.2522    0.2108   -0.2761    0.0610
## 
## Standardized coefficients:
##                 coeff
## FAMILIARITY   -0.0783
## 
## ************ TOTAL, DIRECT, AND INDIRECT EFFECTS OF X ON Y ************
## 
## Total effect of X on Y:
##      effect        se         t         p      LLCI      ULCI      c_ps
##     -0.1076    0.0859   -1.2522    0.2108   -0.2761    0.0610   -0.0783
## 
## Direct effect of X on Y:
##      effect        se         t         p      LLCI      ULCI     c'_ps
##      0.1540    0.0743    2.0735    0.0384    0.0083    0.2997    0.1121
## 
## Indirect effect(s) of X on Y:
##          Effect    BootSE  BootLLCI  BootULCI
## TOTAL   -0.2615    0.0622   -0.3867   -0.1434
## Ind1    -0.1888    0.0350   -0.2584   -0.1223
## Ind2     0.0908    0.0519   -0.0118    0.1946
## Ind3    -0.1636    0.0261   -0.2175   -0.1152
## 
## Partially standardized indirect effect(s) of X on Y:
##          Effect    BootSE  BootLLCI  BootULCI
## TOTAL   -0.1904    0.0454   -0.2814   -0.1041
## Ind1    -0.1375    0.0255   -0.1877   -0.0891
## Ind2     0.0661    0.0375   -0.0085    0.1408
## Ind3    -0.1191    0.0180   -0.1563   -0.0852
## 
## Indirect effect key:
## Ind1 FAMILIARITY    ->    CONNECTED    ->    BEHAVIOR              
## Ind2 FAMILIARITY    ->    SPAx    ->    BEHAVIOR              
## Ind3 FAMILIARITY    ->    CONNECTED    ->    SPAx    ->    BEHAVIOR
## 
## ******************** ANALYSIS NOTES AND ERRORS ************************ 
## 
## Level of confidence for all confidence intervals in output: 95
## 
## Number of bootstraps for percentile bootstrap confidence intervals: 5000
## 
## NOTE: Standardized coefficients for dichotomous or multicategorical X are
##       in partially standardized form.
```

### Explorative Mediation

Analysis:  
- Serial Mediation Model with PROCESS (Model 6)  
- DV: Protective behaviors  
- IV: Familiarity  
- Mediators: Connectedness, Situational pathogen avoidance, Perceived risk of infection

```
## 
## ********************* PROCESS for R Version 4.0.1 ********************* 
##  
##            Written by Andrew F. Hayes, Ph.D.  www.afhayes.com              
##    Documentation available in Hayes (2022). www.guilford.com/p/hayes3   
##  
## *********************************************************************** 
##                    
## Model : 6          
##     Y : BEHAVIOR   
##     X : FAMILIARITY
##    M1 : CONNECTED  
##    M2 : SPAx       
##    M3 : RISK       
## 
## Sample size: 1022
## 
## Random seed: 971458
## 
## 
## *********************************************************************** 
## Outcome Variable: CONNECTED
## 
## Model Summary: 
##           R      R-sq       MSE         F       df1       df2         p
##      0.3968    0.1574    2.9015  190.5758    1.0000 1020.0000    0.0000
## 
## Model: 
##                 coeff        se         t         p      LLCI      ULCI
## constant       0.9230    0.1686    5.4747    0.0000    0.5922    1.2538
## FAMILIARITY    1.4711    0.1066   13.8049    0.0000    1.2620    1.6802
## 
## Standardized coefficients:
##                 coeff
## FAMILIARITY    0.7931
## 
## *********************************************************************** 
## Outcome Variable: SPAx
## 
## Model Summary: 
##           R      R-sq       MSE         F       df1       df2         p
##      0.2543    0.0646    2.0879   35.2128    2.0000 1019.0000    0.0000
## 
## Model: 
##                 coeff        se         t         p      LLCI      ULCI
## constant       5.6345    0.1451   38.8311    0.0000    5.3497    5.9192
## FAMILIARITY    0.1788    0.0985    1.8155    0.0697   -0.0145    0.3720
## CONNECTED     -0.2189    0.0266   -8.2411    0.0000   -0.2710   -0.1668
## 
## Standardized coefficients:
##                 coeff
## FAMILIARITY    0.1198
## CONNECTED     -0.2720
## 
## *********************************************************************** 
## Outcome Variable: RISK
## 
## Model Summary: 
##           R      R-sq       MSE         F       df1       df2         p
##      0.3791    0.1437    2.4256   56.9451    3.0000 1018.0000    0.0000
## 
## Model: 
##                 coeff        se         t         p      LLCI      ULCI
## constant       2.3553    0.2463    9.5636    0.0000    1.8721    2.8386
## FAMILIARITY    0.0763    0.1063    0.7181    0.4729   -0.1323    0.2850
## CONNECTED     -0.0989    0.0296   -3.3435    0.0009   -0.1569   -0.0408
## SPAx           0.3828    0.0338   11.3365    0.0000    0.3165    0.4490
## 
## Standardized coefficients:
##                 coeff
## FAMILIARITY    0.0454
## CONNECTED     -0.1092
## SPAx           0.3400
## 
## *********************************************************************** 
## Outcome Variable: BEHAVIOR
## 
## Model Summary: 
##           R      R-sq       MSE         F       df1       df2         p
##      0.6149    0.3781    1.1778  154.5970    4.0000 1017.0000    0.0000
## 
## Model: 
##                 coeff        se         t         p      LLCI      ULCI
## constant       3.2156    0.1792   17.9482    0.0000    2.8640    3.5672
## FAMILIARITY    0.1500    0.0741    2.0239    0.0432    0.0046    0.2954
## CONNECTED     -0.1232    0.0207   -5.9456    0.0000   -0.1638   -0.0825
## SPAx           0.4879    0.0250   19.5394    0.0000    0.4389    0.5369
## RISK           0.0523    0.0218    2.3945    0.0168    0.0094    0.0952
## 
## Standardized coefficients:
##                 coeff
## FAMILIARITY    0.1092
## CONNECTED     -0.1664
## SPAx           0.5302
## RISK           0.0640
## 
## ************************ TOTAL EFFECT MODEL *************************** 
## Outcome Variable: BEHAVIOR
## 
## Model Summary: 
##           R      R-sq       MSE         F       df1       df2         p
##      0.0392    0.0015    1.8855    1.5681    1.0000 1020.0000    0.2108
## 
## Model: 
##                 coeff        se         t         p      LLCI      ULCI
## constant       5.9795    0.1359   43.9968    0.0000    5.7128    6.2462
## FAMILIARITY   -0.1076    0.0859   -1.2522    0.2108   -0.2761    0.0610
## 
## Standardized coefficients:
##                 coeff
## FAMILIARITY   -0.0783
## 
## ************ TOTAL, DIRECT, AND INDIRECT EFFECTS OF X ON Y ************
## 
## Total effect of X on Y:
##      effect        se         t         p      LLCI      ULCI      c_ps
##     -0.1076    0.0859   -1.2522    0.2108   -0.2761    0.0610   -0.0783
## 
## Direct effect of X on Y:
##      effect        se         t         p      LLCI      ULCI     c'_ps
##      0.1500    0.0741    2.0239    0.0432    0.0046    0.2954    0.1092
## 
## Indirect effect(s) of X on Y:
##          Effect    BootSE  BootLLCI  BootULCI
## TOTAL   -0.2576    0.0615   -0.3782   -0.1376
## Ind1    -0.1812    0.0342   -0.2508   -0.1180
## Ind2     0.0872    0.0502   -0.0127    0.1876
## Ind3     0.0040    0.0062   -0.0075    0.0183
## Ind4    -0.1571    0.0248   -0.2087   -0.1105
## Ind5    -0.0076    0.0041   -0.0166   -0.0009
## Ind6     0.0036    0.0027   -0.0005    0.0100
## Ind7    -0.0064    0.0029   -0.0126   -0.0011
## 
## Partially standardized indirect effect(s) of X on Y:
##          Effect    BootSE  BootLLCI  BootULCI
## TOTAL   -0.1875    0.0449   -0.2779   -0.1009
## Ind1    -0.1319    0.0250   -0.1826   -0.0854
## Ind2     0.0635    0.0363   -0.0093    0.1348
## Ind3     0.0029    0.0045   -0.0055    0.0132
## Ind4    -0.1144    0.0173   -0.1500   -0.0820
## Ind5    -0.0055    0.0030   -0.0121   -0.0007
## Ind6     0.0026    0.0020   -0.0004    0.0072
## Ind7    -0.0047    0.0021   -0.0090   -0.0008
## 
## Indirect effect key:
## Ind1 FAMILIARITY    ->    CONNECTED    ->    BEHAVIOR                            
## Ind2 FAMILIARITY    ->    SPAx    ->    BEHAVIOR                            
## Ind3 FAMILIARITY    ->    RISK    ->    BEHAVIOR                            
## Ind4 FAMILIARITY    ->    CONNECTED    ->    SPAx    ->    BEHAVIOR              
## Ind5 FAMILIARITY    ->    CONNECTED    ->    RISK    ->    BEHAVIOR              
## Ind6 FAMILIARITY    ->    SPAx    ->    RISK    ->    BEHAVIOR              
## Ind7 FAMILIARITY    ->    CONNECTED    ->    SPAx    ->    RISK    ->    BEHAVIOR
## 
## ******************** ANALYSIS NOTES AND ERRORS ************************ 
## 
## Level of confidence for all confidence intervals in output: 95
## 
## Number of bootstraps for percentile bootstrap confidence intervals: 5000
## 
## NOTE: Standardized coefficients for dichotomous or multicategorical X are
##       in partially standardized form.
```

## Study 2

### Mediation Model 3

Analysis:  
- Serial Mediation Model with PROCESS (Model 6)  
- DV: Perceived risk of infection  
- IV: Familiarity  
- Mediators: Connectedness, Situational pathogen avoidance

```
## 
## ********************* PROCESS for R Version 4.0.1 ********************* 
##  
##            Written by Andrew F. Hayes, Ph.D.  www.afhayes.com              
##    Documentation available in Hayes (2022). www.guilford.com/p/hayes3   
##  
## *********************************************************************** 
##                    
## Model : 6          
##     Y : RISK       
##     X : FAMILIARITY
##    M1 : CONNECTED  
##    M2 : SPAx       
## 
## Sample size: 994
## 
## Random seed: 761727
## 
## 
## *********************************************************************** 
## Outcome Variable: CONNECTED
## 
## Model Summary: 
##           R      R-sq       MSE         F       df1       df2         p
##      0.5133    0.2635    2.5227  354.9337    1.0000  992.0000    0.0000
## 
## Model: 
##                 coeff        se         t         p      LLCI      ULCI
## constant       1.0977    0.1588    6.9111    0.0000    0.7860    1.4094
## FAMILIARITY    1.8983    0.1008   18.8397    0.0000    1.7006    2.0960
## 
## Standardized coefficients:
##                 coeff
## FAMILIARITY    1.0262
## 
## *********************************************************************** 
## Outcome Variable: SPAx
## 
## Model Summary: 
##           R      R-sq       MSE         F       df1       df2         p
##      0.1877    0.0352    2.5747   18.0999    2.0000  991.0000    0.0000
## 
## Model: 
##                 coeff        se         t         p      LLCI      ULCI
## constant       4.9957    0.1643   30.4099    0.0000    4.6734    5.3181
## FAMILIARITY    0.0700    0.1186    0.5901    0.5552   -0.1628    0.3028
## CONNECTED     -0.1745    0.0321   -5.4414    0.0000   -0.2375   -0.1116
## 
## Standardized coefficients:
##                 coeff
## FAMILIARITY    0.0429
## CONNECTED     -0.1978
## 
## *********************************************************************** 
## Outcome Variable: RISK
## 
## Model Summary: 
##           R      R-sq       MSE         F       df1       df2         p
##      0.3529    0.1245    2.1613   46.9306    3.0000  990.0000    0.0000
## 
## Model: 
##                 coeff        se         t         p      LLCI      ULCI
## constant       1.7886    0.2093    8.5465    0.0000    1.3779    2.1993
## FAMILIARITY   -0.1294    0.1087   -1.1907    0.2340   -0.3427    0.0839
## CONNECTED      0.0587    0.0298    1.9685    0.0493    0.0002    0.1172
## SPAx           0.3437    0.0291   11.8091    0.0000    0.2866    0.4008
## 
## Standardized coefficients:
##                 coeff
## FAMILIARITY   -0.0825
## CONNECTED      0.0692
## SPAx           0.3575
## 
## ************************ TOTAL EFFECT MODEL *************************** 
## Outcome Variable: RISK
## 
## Model Summary: 
##           R      R-sq       MSE         F       df1       df2         p
##      0.0344    0.0012    2.4608    1.1733    1.0000  992.0000    0.2790
## 
## Model: 
##                 coeff        se         t         p      LLCI      ULCI
## constant       3.5042    0.1569   22.3376    0.0000    3.1964    3.8121
## FAMILIARITY   -0.1078    0.0995   -1.0832    0.2790   -0.3031    0.0875
## 
## Standardized coefficients:
##                 coeff
## FAMILIARITY   -0.0687
## 
## ************ TOTAL, DIRECT, AND INDIRECT EFFECTS OF X ON Y ************
## 
## Total effect of X on Y:
##      effect        se         t         p      LLCI      ULCI      c_ps
##     -0.1078    0.0995   -1.0832    0.2790   -0.3031    0.0875   -0.0687
## 
## Direct effect of X on Y:
##      effect        se         t         p      LLCI      ULCI     c'_ps
##     -0.1294    0.1087   -1.1907    0.2340   -0.3427    0.0839   -0.0825
## 
## Indirect effect(s) of X on Y:
##          Effect    BootSE  BootLLCI  BootULCI
## TOTAL    0.0216    0.0726   -0.1227    0.1621
## Ind1     0.1114    0.0664   -0.0219    0.2442
## Ind2     0.0241    0.0447   -0.0615    0.1120
## Ind3    -0.1139    0.0292   -0.1755   -0.0604
## 
## Partially standardized indirect effect(s) of X on Y:
##          Effect    BootSE  BootLLCI  BootULCI
## TOTAL    0.0138    0.0463   -0.0782    0.1035
## Ind1     0.0710    0.0422   -0.0140    0.1544
## Ind2     0.0153    0.0285   -0.0390    0.0718
## Ind3    -0.0726    0.0186   -0.1122   -0.0385
## 
## Indirect effect key:
## Ind1 FAMILIARITY    ->    CONNECTED    ->    RISK              
## Ind2 FAMILIARITY    ->    SPAx    ->    RISK              
## Ind3 FAMILIARITY    ->    CONNECTED    ->    SPAx    ->    RISK
## 
## ******************** ANALYSIS NOTES AND ERRORS ************************ 
## 
## Level of confidence for all confidence intervals in output: 95
## 
## Number of bootstraps for percentile bootstrap confidence intervals: 5000
## 
## NOTE: Standardized coefficients for dichotomous or multicategorical X are
##       in partially standardized form.
```

### Mediation Model 4

Analysis:  
- Serial Mediation Model with PROCESS (Model 6)  
- DV: protective behaviors  
- IV: Familiarity  
- Mediators: Connectedness, Situational pathogen avoidance

```
## 
## ********************* PROCESS for R Version 4.0.1 ********************* 
##  
##            Written by Andrew F. Hayes, Ph.D.  www.afhayes.com              
##    Documentation available in Hayes (2022). www.guilford.com/p/hayes3   
##  
## *********************************************************************** 
##                    
## Model : 6          
##     Y : BEHAVIOR   
##     X : FAMILIARITY
##    M1 : CONNECTED  
##    M2 : SPAx       
## 
## Sample size: 994
## 
## Random seed: 166283
## 
## 
## *********************************************************************** 
## Outcome Variable: CONNECTED
## 
## Model Summary: 
##           R      R-sq       MSE         F       df1       df2         p
##      0.5133    0.2635    2.5227  354.9337    1.0000  992.0000    0.0000
## 
## Model: 
##                 coeff        se         t         p      LLCI      ULCI
## constant       1.0977    0.1588    6.9111    0.0000    0.7860    1.4094
## FAMILIARITY    1.8983    0.1008   18.8397    0.0000    1.7006    2.0960
## 
## Standardized coefficients:
##                 coeff
## FAMILIARITY    1.0262
## 
## *********************************************************************** 
## Outcome Variable: SPAx
## 
## Model Summary: 
##           R      R-sq       MSE         F       df1       df2         p
##      0.1877    0.0352    2.5747   18.0999    2.0000  991.0000    0.0000
## 
## Model: 
##                 coeff        se         t         p      LLCI      ULCI
## constant       4.9957    0.1643   30.4099    0.0000    4.6734    5.3181
## FAMILIARITY    0.0700    0.1186    0.5901    0.5552   -0.1628    0.3028
## CONNECTED     -0.1745    0.0321   -5.4414    0.0000   -0.2375   -0.1116
## 
## Standardized coefficients:
##                 coeff
## FAMILIARITY    0.0429
## CONNECTED     -0.1978
## 
## *********************************************************************** 
## Outcome Variable: BEHAVIOR
## 
## Model Summary: 
##           R      R-sq       MSE         F       df1       df2         p
##      0.5516    0.3043    1.8861  144.3233    3.0000  990.0000    0.0000
## 
## Model: 
##                 coeff        se         t         p      LLCI      ULCI
## constant       2.1819    0.1955   11.1606    0.0000    1.7982    2.5655
## FAMILIARITY    0.2148    0.1015    2.1153    0.0347    0.0155    0.4140
## CONNECTED     -0.0812    0.0279   -2.9160    0.0036   -0.1359   -0.0266
## SPAx           0.5379    0.0272   19.7847    0.0000    0.4846    0.5913
## 
## Standardized coefficients:
##                 coeff
## FAMILIARITY    0.1307
## CONNECTED     -0.0914
## SPAx           0.5340
## 
## ************************ TOTAL EFFECT MODEL *************************** 
## Outcome Variable: BEHAVIOR
## 
## Model Summary: 
##           R      R-sq       MSE         F       df1       df2         p
##      0.0243    0.0006    2.7039    0.5882    1.0000  992.0000    0.4433
## 
## Model: 
##                 coeff        se         t         p      LLCI      ULCI
## constant       4.6770    0.1644   28.4414    0.0000    4.3543    4.9996
## FAMILIARITY   -0.0800    0.1043   -0.7670    0.4433   -0.2847    0.1247
## 
## Standardized coefficients:
##                 coeff
## FAMILIARITY   -0.0487
## 
## 
## ************ TOTAL, DIRECT, AND INDIRECT EFFECTS OF X ON Y ************
## 
## Total effect of X on Y:
##      effect        se         t         p      LLCI      ULCI      c_ps
##     -0.0800    0.1043   -0.7670    0.4433   -0.2847    0.1247   -0.0487
## 
## Direct effect of X on Y:
##      effect        se         t         p      LLCI      ULCI     c'_ps
##      0.2148    0.1015    2.1153    0.0347    0.0155    0.4140    0.1306
## 
## Indirect effect(s) of X on Y:
##          Effect    BootSE  BootLLCI  BootULCI
## TOTAL   -0.2948    0.0814   -0.4595   -0.1398
## Ind1    -0.1542    0.0596   -0.2763   -0.0423
## Ind2     0.0377    0.0689   -0.0957    0.1738
## Ind3    -0.1782    0.0409   -0.2615   -0.0998
## 
## Partially standardized indirect effect(s) of X on Y:
##          Effect    BootSE  BootLLCI  BootULCI
## TOTAL   -0.1793    0.0494   -0.2781   -0.0856
## Ind1    -0.0938    0.0362   -0.1682   -0.0260
## Ind2     0.0229    0.0419   -0.0579    0.1062
## Ind3    -0.1084    0.0247   -0.1583   -0.0609
## 
## Indirect effect key:
## Ind1 FAMILIARITY    ->    CONNECTED    ->    BEHAVIOR              
## Ind2 FAMILIARITY    ->    SPAx    ->    BEHAVIOR              
## Ind3 FAMILIARITY    ->    CONNECTED    ->    SPAx    ->    BEHAVIOR
## 
## ******************** ANALYSIS NOTES AND ERRORS ************************ 
## 
## Level of confidence for all confidence intervals in output: 95
## 
## Number of bootstraps for percentile bootstrap confidence intervals: 5000
## 
## NOTE: Standardized coefficients for dichotomous or multicategorical X are
##       in partially standardized form.
```

### Explorative Mediation

Analysis:  
- Serial Mediation Model with PROCESS (Model 6)  
- DV: protective behaviors  
- IV: Familiarity  
- Mediators: Connectedness, Situational pathogen avoidance, perceived risk of infection

```
## 
## ********************* PROCESS for R Version 4.0.1 ********************* 
##  
##            Written by Andrew F. Hayes, Ph.D.  www.afhayes.com              
##    Documentation available in Hayes (2022). www.guilford.com/p/hayes3   
##  
## *********************************************************************** 
##                    
## Model : 6          
##     Y : BEHAVIOR   
##     X : FAMILIARITY
##    M1 : CONNECTED  
##    M2 : SPAx       
##    M3 : RISK       
## 
## Sample size: 994
## 
## Random seed: 626761
## 
## 
## *********************************************************************** 
## Outcome Variable: CONNECTED
## 
## Model Summary: 
##           R      R-sq       MSE         F       df1       df2         p
##      0.5133    0.2635    2.5227  354.9337    1.0000  992.0000    0.0000
## 
## Model: 
##                 coeff        se         t         p      LLCI      ULCI
## constant       1.0977    0.1588    6.9111    0.0000    0.7860    1.4094
## FAMILIARITY    1.8983    0.1008   18.8397    0.0000    1.7006    2.0960
## 
## Standardized coefficients:
##                 coeff
## FAMILIARITY    1.0262
## 
## *********************************************************************** 
## Outcome Variable: SPAx
## 
## Model Summary: 
##           R      R-sq       MSE         F       df1       df2         p
##      0.1877    0.0352    2.5747   18.0999    2.0000  991.0000    0.0000
## 
## Model: 
##                 coeff        se         t         p      LLCI      ULCI
## constant       4.9957    0.1643   30.4099    0.0000    4.6734    5.3181
## FAMILIARITY    0.0700    0.1186    0.5901    0.5552   -0.1628    0.3028
## CONNECTED     -0.1745    0.0321   -5.4414    0.0000   -0.2375   -0.1116
## 
## Standardized coefficients:
##                 coeff
## FAMILIARITY    0.0429
## CONNECTED     -0.1978
## 
## *********************************************************************** 
## Outcome Variable: RISK
## 
## Model Summary: 
##           R      R-sq       MSE         F       df1       df2         p
##      0.3529    0.1245    2.1613   46.9306    3.0000  990.0000    0.0000
## 
## Model: 
##                 coeff        se         t         p      LLCI      ULCI
## constant       1.7886    0.2093    8.5465    0.0000    1.3779    2.1993
## FAMILIARITY   -0.1294    0.1087   -1.1907    0.2340   -0.3427    0.0839
## CONNECTED      0.0587    0.0298    1.9685    0.0493    0.0002    0.1172
## SPAx           0.3437    0.0291   11.8091    0.0000    0.2866    0.4008
## 
## Standardized coefficients:
##                 coeff
## FAMILIARITY   -0.0825
## CONNECTED      0.0692
## SPAx           0.3575
## 
## *********************************************************************** 
## Outcome Variable: BEHAVIOR
## 
## Model Summary: 
##           R      R-sq       MSE         F       df1       df2         p
##      0.5643    0.3185    1.8495  115.5351    4.0000  989.0000    0.0000
## 
## Model: 
##                 coeff        se         t         p      LLCI      ULCI
## constant       1.9432    0.2006    9.6867    0.0000    1.5496    2.3369
## FAMILIARITY    0.2321    0.1006    2.3062    0.0213    0.0346    0.4295
## CONNECTED     -0.0891    0.0276   -3.2224    0.0013   -0.1433   -0.0348
## SPAx           0.4921    0.0288   17.1107    0.0000    0.4356    0.5485
## RISK           0.1334    0.0294    4.5386    0.0000    0.0757    0.1911
## 
## Standardized coefficients:
##                 coeff
## FAMILIARITY    0.1412
## CONNECTED     -0.1003
## SPAx           0.4885
## RISK           0.1273
## 
## ************************ TOTAL EFFECT MODEL *************************** 
## Outcome Variable: BEHAVIOR
## 
## Model Summary: 
##           R      R-sq       MSE         F       df1       df2         p
##      0.0243    0.0006    2.7039    0.5882    1.0000  992.0000    0.4433
## 
## Model: 
##                 coeff        se         t         p      LLCI      ULCI
## constant       4.6770    0.1644   28.4414    0.0000    4.3543    4.9996
## FAMILIARITY   -0.0800    0.1043   -0.7670    0.4433   -0.2847    0.1247
## 
## Standardized coefficients:
##                 coeff
## FAMILIARITY   -0.0487
## 
## 
## ************ TOTAL, DIRECT, AND INDIRECT EFFECTS OF X ON Y ************
## 
## Total effect of X on Y:
##      effect        se         t         p      LLCI      ULCI      c_ps
##     -0.0800    0.1043   -0.7670    0.4433   -0.2847    0.1247   -0.0487
## 
## Direct effect of X on Y:
##      effect        se         t         p      LLCI      ULCI     c'_ps
##      0.2321    0.1006    2.3062    0.0213    0.0346    0.4295    0.1412
## 
## Indirect effect(s) of X on Y:
##          Effect    BootSE  BootLLCI  BootULCI
## TOTAL   -0.3121    0.0828   -0.4826   -0.1536
## Ind1    -0.1691    0.0592   -0.2911   -0.0565
## Ind2     0.0344    0.0643   -0.0944    0.1603
## Ind3    -0.0173    0.0159   -0.0513    0.0113
## Ind4    -0.1630    0.0386   -0.2439   -0.0922
## Ind5     0.0149    0.0096   -0.0016    0.0359
## Ind6     0.0032    0.0061   -0.0089    0.0154
## Ind7    -0.0152    0.0052   -0.0263   -0.0062
## 
## Partially standardized indirect effect(s) of X on Y:
##          Effect    BootSE  BootLLCI  BootULCI
## TOTAL   -0.1898    0.0502   -0.2922   -0.0937
## Ind1    -0.1029    0.0359   -0.1759   -0.0344
## Ind2     0.0209    0.0391   -0.0573    0.0966
## Ind3    -0.0105    0.0097   -0.0312    0.0069
## Ind4    -0.0992    0.0233   -0.1471   -0.0561
## Ind5     0.0090    0.0058   -0.0010    0.0218
## Ind6     0.0020    0.0037   -0.0054    0.0094
## Ind7    -0.0092    0.0031   -0.0160   -0.0038
## 
## Indirect effect key:
## Ind1 FAMILIARITY    ->    CONNECTED    ->    BEHAVIOR                            
## Ind2 FAMILIARITY    ->    SPAx    ->    BEHAVIOR                            
## Ind3 FAMILIARITY    ->    RISK    ->    BEHAVIOR                            
## Ind4 FAMILIARITY    ->    CONNECTED    ->    SPAx    ->    BEHAVIOR              
## Ind5 FAMILIARITY    ->    CONNECTED    ->    RISK    ->    BEHAVIOR              
## Ind6 FAMILIARITY    ->    SPAx    ->    RISK    ->    BEHAVIOR              
## Ind7 FAMILIARITY    ->    CONNECTED    ->    SPAx    ->    RISK    ->    BEHAVIOR
## 
## ******************** ANALYSIS NOTES AND ERRORS ************************ 
## 
## Level of confidence for all confidence intervals in output: 95
## 
## Number of bootstraps for percentile bootstrap confidence intervals: 5000
## 
## NOTE: Standardized coefficients for dichotomous or multicategorical X are
##       in partially standardized form.
```

### ANOVA

Analysis:  
- Analysis of variance  
- DV: Situational pathogen avoidance  
- IVs: COVID-19 risk status (vaccinated vs. not vaccinated but tested vs. no information) and Familiarity (friend vs. stranger) and interaction COVID-19 risk status x Familiarity

```
## Anova Table (Type III tests)
## 
## Response: SPAx
##                     Sum Sq  Df  F value Pr(>F)    
## (Intercept)         377.04   1 142.1181 <2e-16 ***
## FAMILIARITY           6.75   1   2.5436 0.1111    
## STATUS                1.14   1   0.4284 0.5129    
## FAMILIARITY:STATUS    1.25   1   0.4727 0.4919    
## Residuals          2626.46 990                    
## ---
## Signif. codes:  0 '***' 0.001 '**' 0.01 '*' 0.05 '.' 0.1 ' ' 1
```

```
## 
##  Shapiro-Wilk normality test
## 
## data:  PARTY2$SPAx
## W = 0.96697, p-value = 3.093e-14
```

## Appendix

### Mediation Model 3 controlled for own vaccination status

Analysis:  
- Serial Mediation Model with PROCESS (Model 6)  
- DV: Perceived risk of infection  
- IV: Familiarity  
- CV: Own vaccination status - Mediators: Connectedness, Situational pathogen avoidance

```
## 
## ********************* PROCESS for R Version 4.0.1 ********************* 
##  
##            Written by Andrew F. Hayes, Ph.D.  www.afhayes.com              
##    Documentation available in Hayes (2022). www.guilford.com/p/hayes3   
##  
## *********************************************************************** 
##                    
## Model : 6          
##     Y : RISK       
##     X : FAMILIARITY
##    M1 : CONNECTED  
##    M2 : SPAx       
## 
## Covariates: 
##        OWN_STATUS
## 
## Sample size: 994
## 
## Random seed: 432724
## 
## 
## *********************************************************************** 
## Outcome Variable: CONNECTED
## 
## Model Summary: 
##           R      R-sq       MSE         F       df1       df2         p
##      0.5166    0.2669    2.5136  180.4016    2.0000  991.0000    0.0000
## 
## Model: 
##                 coeff        se         t         p      LLCI      ULCI
## constant       0.6545    0.2607    2.5101    0.0122    0.1428    1.1661
## FAMILIARITY    1.9057    0.1006   18.9363    0.0000    1.7082    2.1032
## OWN_STATUS     0.4007    0.1871    2.1416    0.0325    0.0335    0.7679
## 
## Standardized coefficients:
##                 coeff
## FAMILIARITY    1.0302
## OWN_STATUS     0.0583
## 
## *********************************************************************** 
## Outcome Variable: SPAx
## 
## Model Summary: 
##           R      R-sq       MSE         F       df1       df2         p
##      0.1882    0.0354    2.5768   12.1178    3.0000  990.0000    0.0000
## 
## Model: 
##                 coeff        se         t         p      LLCI      ULCI
## constant       4.9068    0.2648   18.5284    0.0000    4.3871    5.4264
## FAMILIARITY    0.0733    0.1189    0.6163    0.5379   -0.1601    0.3066
## CONNECTED     -0.1755    0.0322   -5.4557    0.0000   -0.2386   -0.1124
## OWN_STATUS     0.0814    0.1899    0.4286    0.6683   -0.2913    0.4540
## 
## Standardized coefficients:
##                 coeff
## FAMILIARITY    0.0449
## CONNECTED     -0.1989
## OWN_STATUS     0.0134
## 
## *********************************************************************** 
## Outcome Variable: RISK
## 
## Model Summary: 
##           R      R-sq       MSE         F       df1       df2         p
##      0.3532    0.1248    2.1629   35.2474    4.0000  989.0000    0.0000
## 
## Model: 
##                 coeff        se         t         p      LLCI      ULCI
## constant       1.6858    0.2816    5.9874    0.0000    1.1333    2.2384
## FAMILIARITY   -0.1256    0.1090   -1.1525    0.2494   -0.3394    0.0882
## CONNECTED      0.0576    0.0299    1.9254    0.0545   -0.0011    0.1163
## SPAx           0.3435    0.0291   11.7963    0.0000    0.2863    0.4006
## OWN_STATUS     0.0949    0.1740    0.5456    0.5855   -0.2465    0.4364
## 
## Standardized coefficients:
##                 coeff
## FAMILIARITY   -0.0801
## CONNECTED      0.0679
## SPAx           0.3573
## OWN_STATUS     0.0163
## 
## ************************ TOTAL EFFECT MODEL *************************** 
## Outcome Variable: RISK
## 
## Model Summary: 
##           R      R-sq       MSE         F       df1       df2         p
##      0.0402    0.0016    2.4622    0.8026    2.0000  991.0000    0.4484
## 
## Model: 
##                 coeff        se         t         p      LLCI      ULCI
## constant       3.3695    0.2581   13.0574    0.0000    2.8631    3.8759
## FAMILIARITY   -0.1055    0.0996   -1.0596    0.2896   -0.3010    0.0899
## OWN_STATUS     0.1218    0.1852    0.6577    0.5109   -0.2416    0.4852
## 
## Standardized coefficients:
##                 coeff
## FAMILIARITY   -0.0672
## OWN_STATUS     0.0209
## 
## 
## ************ TOTAL, DIRECT, AND INDIRECT EFFECTS OF X ON Y ************
## 
## Total effect of X on Y:
##      effect        se         t         p      LLCI      ULCI      c_ps
##     -0.1055    0.0996   -1.0596    0.2896   -0.3010    0.0899   -0.0673
## 
## Direct effect of X on Y:
##      effect        se         t         p      LLCI      ULCI     c'_ps
##     -0.1256    0.1090   -1.1525    0.2494   -0.3394    0.0882   -0.0800
## 
## Indirect effect(s) of X on Y:
##          Effect    BootSE  BootLLCI  BootULCI
## TOTAL    0.0200    0.0723   -0.1217    0.1622
## Ind1     0.1097    0.0671   -0.0202    0.2410
## Ind2     0.0252    0.0439   -0.0587    0.1143
## Ind3    -0.1149    0.0290   -0.1753   -0.0627
## 
## Partially standardized indirect effect(s) of X on Y:
##          Effect    BootSE  BootLLCI  BootULCI
## TOTAL    0.0128    0.0461   -0.0774    0.1033
## Ind1     0.0699    0.0427   -0.0127    0.1528
## Ind2     0.0160    0.0280   -0.0373    0.0731
## Ind3    -0.0732    0.0185   -0.1124   -0.0399
## 
## Indirect effect key:
## Ind1 FAMILIARITY    ->    CONNECTED    ->    RISK              
## Ind2 FAMILIARITY    ->    SPAx    ->    RISK              
## Ind3 FAMILIARITY    ->    CONNECTED    ->    SPAx    ->    RISK
## 
## ******************** ANALYSIS NOTES AND ERRORS ************************ 
## 
## Level of confidence for all confidence intervals in output: 95
## 
## Number of bootstraps for percentile bootstrap confidence intervals: 5000
## 
## NOTE: Standardized coefficients for dichotomous or multicategorical X are
##       in partially standardized form.
```

### Mediation Model 4 controlled for own vaccination status

Analysis:  
- Serial Mediation Model with PROCESS (Model 6)  
- DV: Protective behaviors  
- IV: Familiarity  
- CV: Own vaccination status - Mediators: Connectedness, Situational pathogen avoidance

```
## 
## ********************* PROCESS for R Version 4.0.1 ********************* 
##  
##            Written by Andrew F. Hayes, Ph.D.  www.afhayes.com              
##    Documentation available in Hayes (2022). www.guilford.com/p/hayes3   
##  
## *********************************************************************** 
##                    
## Model : 6          
##     Y : BEHAVIOR   
##     X : FAMILIARITY
##    M1 : CONNECTED  
##    M2 : SPAx       
## 
## Covariates: 
##        OWN_STATUS
## 
## Sample size: 994
## 
## Random seed: 320064
## 
## 
## *********************************************************************** 
## Outcome Variable: CONNECTED
## 
## Model Summary: 
##           R      R-sq       MSE         F       df1       df2         p
##      0.5166    0.2669    2.5136  180.4016    2.0000  991.0000    0.0000
## 
## Model: 
##                 coeff        se         t         p      LLCI      ULCI
## constant       0.6545    0.2607    2.5101    0.0122    0.1428    1.1661
## FAMILIARITY    1.9057    0.1006   18.9363    0.0000    1.7082    2.1032
## OWN_STATUS     0.4007    0.1871    2.1416    0.0325    0.0335    0.7679
## 
## Standardized coefficients:
##                 coeff
## FAMILIARITY    1.0302
## OWN_STATUS     0.0583
## 
## *********************************************************************** 
## Outcome Variable: SPAx
## 
## Model Summary: 
##           R      R-sq       MSE         F       df1       df2         p
##      0.1882    0.0354    2.5768   12.1178    3.0000  990.0000    0.0000
## 
## Model: 
##                 coeff        se         t         p      LLCI      ULCI
## constant       4.9068    0.2648   18.5284    0.0000    4.3871    5.4264
## FAMILIARITY    0.0733    0.1189    0.6163    0.5379   -0.1601    0.3066
## CONNECTED     -0.1755    0.0322   -5.4557    0.0000   -0.2386   -0.1124
## OWN_STATUS     0.0814    0.1899    0.4286    0.6683   -0.2913    0.4540
## 
## Standardized coefficients:
##                 coeff
## FAMILIARITY    0.0449
## CONNECTED     -0.1989
## OWN_STATUS     0.0134
## 
## *********************************************************************** 
## Outcome Variable: BEHAVIOR
## 
## Model Summary: 
##           R      R-sq       MSE         F       df1       df2         p
##      0.5517    0.3044    1.8877  108.1894    4.0000  989.0000    0.0000
## 
## Model: 
##                 coeff        se         t         p      LLCI      ULCI
## constant       2.2515    0.2630    8.5594    0.0000    1.7353    2.7677
## FAMILIARITY    0.2122    0.1018    2.0843    0.0374    0.0124    0.4119
## CONNECTED     -0.0805    0.0279   -2.8804    0.0041   -0.1353   -0.0256
## SPAx           0.5381    0.0272   19.7798    0.0000    0.4847    0.5914
## OWN_STATUS    -0.0644    0.1625   -0.3959    0.6922   -0.3833    0.2546
## 
## Standardized coefficients:
##                 coeff
## FAMILIARITY    0.1291
## CONNECTED     -0.0906
## SPAx           0.5342
## OWN_STATUS    -0.0105
## 
## ************************ TOTAL EFFECT MODEL *************************** 
## Outcome Variable: BEHAVIOR
## 
## Model Summary: 
##           R      R-sq       MSE         F       df1       df2         p
##      0.0285    0.0008    2.7061    0.4029    2.0000  991.0000    0.6685
## 
## Model: 
##                 coeff        se         t         p      LLCI      ULCI
## constant       4.7772    0.2705   17.6589    0.0000    4.2464    5.3081
## FAMILIARITY   -0.0817    0.1044   -0.7823    0.4342   -0.2866    0.1232
## OWN_STATUS    -0.0907    0.1941   -0.4669    0.6407   -0.4716    0.2903
## 
## Standardized coefficients:
##                 coeff
## FAMILIARITY   -0.0497
## OWN_STATUS    -0.0148
## 
## 
## ************ TOTAL, DIRECT, AND INDIRECT EFFECTS OF X ON Y ************
## 
## Total effect of X on Y:
##      effect        se         t         p      LLCI      ULCI      c_ps
##     -0.0817    0.1044   -0.7823    0.4342   -0.2866    0.1232   -0.0497
## 
## Direct effect of X on Y:
##      effect        se         t         p      LLCI      ULCI     c'_ps
##      0.2122    0.1018    2.0843    0.0374    0.0124    0.4119    0.1291
## 
## Indirect effect(s) of X on Y:
##          Effect    BootSE  BootLLCI  BootULCI
## TOTAL   -0.2939    0.0821   -0.4580   -0.1338
## Ind1    -0.1534    0.0595   -0.2731   -0.0395
## Ind2     0.0394    0.0690   -0.0923    0.1755
## Ind3    -0.1799    0.0413   -0.2623   -0.0998
## 
## Partially standardized indirect effect(s) of X on Y:
##          Effect    BootSE  BootLLCI  BootULCI
## TOTAL   -0.1787    0.0498   -0.2778   -0.0812
## Ind1    -0.0933    0.0361   -0.1658   -0.0244
## Ind2     0.0240    0.0419   -0.0563    0.1069
## Ind3    -0.1094    0.0249   -0.1589   -0.0614
## 
## Indirect effect key:
## Ind1 FAMILIARITY    ->    CONNECTED    ->    BEHAVIOR              
## Ind2 FAMILIARITY    ->    SPAx    ->    BEHAVIOR              
## Ind3 FAMILIARITY    ->    CONNECTED    ->    SPAx    ->    BEHAVIOR
## 
## ******************** ANALYSIS NOTES AND ERRORS ************************ 
## 
## Level of confidence for all confidence intervals in output: 95
## 
## Number of bootstraps for percentile bootstrap confidence intervals: 5000
## 
## NOTE: Standardized coefficients for dichotomous or multicategorical X are
##       in partially standardized form.
```

### MANOVA 1

Analysis:  
- Multivariant Analysis of variance  
- DVs: Perceived risk of infection and Protective behavior  
- IVs: COVID-19 risk status (vaccinated vs. not vaccinated but tested vs. no information) and Familiarity (friend vs. stranger) and interaction COVID-19 risk status x Familiarity

```
## 
## Type III MANOVA Tests: Pillai test statistic
##                    Df test stat approx F num Df den Df Pr(>F)    
## (Intercept)         1  0.126557   71.650      2    989 <2e-16 ***
## STATUS              1  0.000247    0.122      2    989 0.8851    
## FAMILIARITY         1  0.001123    0.556      2    989 0.5738    
## STATUS:FAMILIARITY  1  0.001038    0.514      2    989 0.5984    
## ---
## Signif. codes:  0 '***' 0.001 '**' 0.01 '*' 0.05 '.' 0.1 ' ' 1
```

### MANOVA 1a

Analysis:  
- Multivariant Analysis of variance  
- DVs: Perceived risk of infection and protective behavior  
- IVs: Dummy vac (vaccinated vs. no information), Dummy test (not vaccinated but tested vs. no information), and Familiarity (friend vs. stranger) and interactions Dummy vac x Familiarity and Dummy test x Familiarity

```
## 
## Type III MANOVA Tests: Pillai test statistic
##                        Df test stat approx F num Df den Df Pr(>F)    
## (Intercept)             1  0.240981  156.681      2    987 <2e-16 ***
## DUMMY_VAC               1  0.000197    0.097      2    987 0.9073    
## DUMMY_TEST              1  0.001661    0.821      2    987 0.4402    
## FAMILIARITY             1  0.000405    0.200      2    987 0.8189    
## DUMMY_VAC:FAMILIARITY   1  0.001022    0.505      2    987 0.6037    
## DUMMY_TEST:FAMILIARITY  1  0.001133    0.560      2    987 0.5715    
## ---
## Signif. codes:  0 '***' 0.001 '**' 0.01 '*' 0.05 '.' 0.1 ' ' 1
```

### Testing requirements MANOVA

```
## 
##  Shapiro-Wilk normality test
## 
## data:  PARTY2$RISK
## W = 0.93568, p-value < 2.2e-16
```

```
## 
##  Shapiro-Wilk normality test
## 
## data:  PARTY2$BEHAVIOR
## W = 0.95986, p-value = 6.934e-16
```

### MANOVA 2 Analysis:  
- Multivariant Analysis of variance  
- DVs: Perceived risk of infection and Protective behavior  
- IVs: COVID-19 risk status (vaccinated vs. not vaccinated but tested vs. no information)

```
## 
## Type III MANOVA Tests: Pillai test statistic
##             Df test stat approx F num Df den Df  Pr(>F)    
## (Intercept)  1   0.55245   611.63      2    991 < 2e-16 ***
## STATUS       1   0.00773     3.86      2    991 0.02138 *  
## ---
## Signif. codes:  0 '***' 0.001 '**' 0.01 '*' 0.05 '.' 0.1 ' ' 1
```

```
## Anova Table (Type III tests)
## 
## Response: RISK
##              Sum Sq  Df  F value Pr(>F)    
## (Intercept) 1551.07   1 629.6793 <2e-16 ***
## STATUS         0.45   1   0.1818 0.6699    
## Residuals   2443.57 992                    
## ---
## Signif. codes:  0 '***' 0.001 '**' 0.01 '*' 0.05 '.' 0.1 ' ' 1
```

```
## Anova Table (Type III tests)
## 
## Response: BEHAVIOR
##             Sum Sq  Df  F value  Pr(>F)    
## (Intercept) 2536.5   1 944.6805 < 2e-16 ***
## STATUS        20.3   1   7.5619 0.00607 ** 
## Residuals   2663.6 992                     
## ---
## Signif. codes:  0 '***' 0.001 '**' 0.01 '*' 0.05 '.' 0.1 ' ' 1
```
